# Mineral and Ester Nanofluids as Dielectric Cooling Liquid for Power Transformers

**DOI:** 10.3390/nano12152723

**Published:** 2022-08-08

**Authors:** Cristian Olmo, Cristina Méndez, Pedro J. Quintanilla, Félix Ortiz, Carlos J. Renedo, Alfredo Ortiz

**Affiliations:** Department of Electrical and Energy Engineering, Universidad de Cantabria, 39005 Santander, Spain

**Keywords:** transformer oil, thermal-dielectric nanofluid, preparation, characteristics, applicability

## Abstract

Amidst the new techniques facing the improvement of cooling and insulating efficiency and the design of electric transformers, constrained by the current technologies, one of the more promising is the substitution of traditional dielectric oils for nanofluids. Research on nanofluids for their application in transformers as a coolant and dielectric medium have been performed during the last two decades and continue today. This review tries to collect and analyze the available information in this field and to offer it already dissected to researchers, focusing on the preparation methods and how nanoparticles affect the main properties of the base fluids. Here we also addressed the influence of different parameters as particle characteristics or environmental conditions in nanofluids performance, the evolution with time of the measured properties, or the neighboring relationship of nanofluids with other transformer components. In this sense, the most reviewed articles reflect enhancements of thermal conductivity or dielectric strength, as well as an improvement of time evolution of these properties, with respect to those that are found in base fluids, and, also, a better interaction between these nanofluids and dielectric cellulosics. Thus, the use of dielectric nanofluids in transformers may allow these machines to work safer or over their design parameters, reducing the risk of failure of the electrical networks and enhancing their life expectancy. Nevertheless, these advantages will not be useful unless a proper stability of nanofluids is ensured, which is achieved in a small part of revised articles. A compendium of the preparation methodology with this aim is proposed, to be checked in future works.

## 1. Introduction

Beyond their potential applications in the generation and storage of energy, nanoparticles may play an important role in energy transmission systems. Current electric systems need to adapt to meet the challenges resulting from the increase in electric transportation, which will require grids with higher capacity and reliability. The near-future needs in terms of design and capabilities of power transformers, key grid elements, and attempts to increase the life expectancy and efficiency of equipment have boosted the development of techniques to improve the cooling and isolation of these machines, such as the application of dielectric oil-based nanofluids. Proposed originally in 1998 by Segal et al. [1], who started research in this area, the main idea was to translate the supposed advantages of nanofluids as a coolant medium (pointed out a few years earlier by Choi et al. [2]) to the cooling systems of high power transformers. This is achieved by the addition and dispersion of low quantities of nanoparticles in traditional transformer mineral oils.

Theoretically, the larger thermal conductivity of nanoparticles in comparison to the fluid may lead to an improvement in cooling and, therefore, a reduction in hot-spots and the mean temperatures of the copper wires. This provides an opportunity for the power enhancement of this equipment, while protecting the components from thermal failure more effectively. Nevertheless, to ensure that the use of nanofluids on transformers is viable, other properties must be considered.

Nanoparticles have unique properties that arise from scale effects. Due to their size, their surface/volume (S/V) ratio is much larger than bulk materials. Consequently, the relative presence of atoms and their availability for surface interactions with surrounding media is higher, providing them with special properties. This is why, over the last two decades, different researchers have put their efforts into the study of dielectric nanofluids. They have investigated properties that are related to thermal transport and cooling, such as thermal conductivity, viscosity, convective coefficient, and specific heat; electric isolation capabilities of the fluid (mainly dielectric strength); and those that define the relationship between the fluid and other components of the system or how the materials evolve over time under different stresses and environments, such as stability, aging resilience, and the degree of polymerization of the dielectric cellulosic.

The results were very often positive and unexpected. This encouraged further investigation, with the aim of obtaining a suitable thermal-dielectric nanofluid. This article attempts to collate and summarize these works. This information is presented to the readers introducing both a general view of the current development of the dielectric nanofluids for transformers or a specific revelation in one of the multiple aspects of this topic. We first focus on the preparation methods that are found in dielectric-nanofluid research and how they condition the stability of the resulting dispersions. A great variety of preparation methodologies and nanofluid components (base oils, particles, and treatments) were found in the papers that we reviewed. An attempt to describe standardized methods that can be applied to produce successful dielectric nanofluids with long-term stability has been carried out. The subsequent sections point to the critical properties of the prepared nanofluids, and to the essential aspects which the application of dielectric nanofluids rely on. In this sense, the focus has been on the cooling capacities and the dielectric properties of nanofluids. The conditions that are required for their optimization have been approached. Their evolution with time and how the nanoparticle presence affects other transformer components have also been analyzed.

## 2. Preparation and Stability of Dielectric Nanofluids

### 2.1. Nanoparticle Characteristics

The idea of using nanoparticles in fluids first arose from the need to avoid the disadvantages that microfluids (those with a portion of the dispersed particles in the microscale) presented as coolants [2]. These disadvantages included instability, the sedimentation of the solid fraction, resulting in the loss of any improvement in the base fluid properties, and undesirable consequences such as abrasion of the pipes and an increase in the pumping power requirements due to a penalty on pressure drop. It must be considered that the current mean time of residence of a traditional dielectric fluid in a transformer that is under use is several years [3,4]. So, the aim is to find nanofluid dispersions that last as long as possible, with reduced maintenance and economical operation of installations.

The disadvantages of microfluids are mainly due to the size and concentration of the particles that are used. The stability of the particle dispersion depends on these parameters, as they predetermine if gravity, buoyancy forces, and the surface interactions with the fluid are in equilibrium. A greater mean size or a lower S/V ratio leads to a reduction in the surface interactions and to greater gravitational forces, promoting sedimentation [5]. Similarly, a higher concentration of particles promotes aggregation as successful contacts between particles rises. Moreover, it increases abrasion and the pumping requirements.

Thus, a fluid with fewer and smaller particles may have an optimized number and strength of interactions among the different components, reaching a new equilibrium of forces that keeps the dispersion stable. At the same time, due to the low concentration of particles that are used, this nanofluid may be less abrasive and would have a viscosity and density that is close to the base fluid, resulting in a more suitable fluid for use in cooling circuits. All these provisions have been considered during the preparation of dielectric nanofluids.

By definition, nanoparticles have a maximum size of 100 nm in any direction [6], but most of the references we examined were carried out with mean sizes below 20 nm (63%), or with maximum sizes below 50 nm (78%), in order to increase the S/V ratio. The reviewed papers are classified as a function of the size of the added particles in Table 1.

As mentioned earlier, the stability of the particle dispersion depends on the particle size, and this parameter grows with time due to aggregation. This phenomenon occurs when particles collide with each other, and it becomes more frequent as the particle concentration increases. This, together with the consequences of over viscosity that were discussed earlier, hinders the use of elevated concentrations, even though it is supposed that the beneficial effects of nanoparticles would increase with concentration.

A compromise solution was pursued. A volume fraction below 1% is recommended [81], in which is a mass fraction of 5.9% or 52 g/L for magnetite in mineral oil, and 4.47% or 39 g/L for titania in mineral oil. Table 2 summarizes the concentration ranges of the tested nanofluids, very often only ten to one hundred times the trace concentration, or even less. Approximately 77% of the reviewed papers presented concentrations that were less than the recommended values.

Dielectric cooling fluids for transformers are organic chain compounds that are obtained from petroleum and vegetal sources, as is the case for mineral oils and ester-based fluids, respectively. These are mainly non-polar and hydrophobic molecules, so their interactions with nanoparticle surfaces are not electrostatic such as those in watery colloids [5]. Beyond size and concentration, the surface characteristics and interactions with the surrounding medium must be considered, along with other aspects, both internal and external, that can affect stability. According to the references, this includes the shape and behavior under high temperatures and magnetic fields. Thus, nanoparticles that are added to dielectric fluids should necessarily gather chemical stability under the expected work conditions and tend to interact with the base fluid.

Since the beginning of these investigations, the most common nanoparticles have been metal oxides, which are very stable compounds, specifically magnetite (Fe_2_O_3_) and other iron species (38% of thermal-dielectric nanofluids) and titania (TiO_2_, 20%). Alumina (Al_2_O_3_) and silica (SiO_2_) are also common (approximately 10% each), with other metallic oxides (10%), and all other nanoparticles (12%) occurring less frequently. Some examples can be found using mixtures of the previous oxides [128], or pure metal nanoparticles, which tend naturally to oxidize. Over time, as they become more frequent in other fields of nanotechnology, different nanostructures such as graphene, fullerene, and carbon nanotubes (CNT) have been used with the expectation that their organic composition will make them suitable for dispersions with long-term stability. Table 3 gives a breakdown of the different nanoparticles that were found in the works that we reviewed, specifically dedicated to thermal-dielectric analysis.

From the point of view of their properties, mainly based on their respective bulk materials, these nanoparticles fulfil the condition of having thermal conductivities that are several times greater than the dielectric base fluids, approximately ten times greater for magnetite, and more than ten thousand times greater for diamond and carbon nanotubes (Table 4) [5,26,30,82,88,130].

Nanoparticles can also be classified according to their electric conductivity as conductive, semiconductive, and insulating [35], as Table 5 shows [82,130,141]. Contrary to the general belief, the addition of resistive nanoparticles is not required to achieve dielectric nanofluids.

Considering shape, it seems that nanoparticles with an elongated shape, such as nanotubes with high length/diameter (L/D) aspect ratios tend easily to aggregate [27,61,142]. A clear majority of the nanoparticles that were used in the reviewed research were described as spherical or quasi-spherical, especially the metallic oxides, but there are examples with other geometries, such as sheets [115].

Considering other aspects, it is very important to condition the nanofluid component selection for the expected application and the associated environmental conditions, trying to fulfil the requirements for the physical integrity of the particles over time, suitable behavior, and a beneficial relationship with the base fluid. It appears that coolant-nanofluid dispersion could be improved by controlling the operating temperature. The Brownian movement of particles in fluid is enhanced as the temperature increases, so perdurable interactions between the particles become less probable, hindering aggregation, as noticed in [9,143]. Nevertheless, it is compulsory for these nanoparticles to withstand such conditions. Magnetic nanoparticles can be affected by external magnetic fields; as they line up according to the field direction, their Brownian movement is constrained, and their interactions get stronger [33,99,100].

### 2.2. Synthesis and Production Methods of Nanoparticles

**Figure 1:**
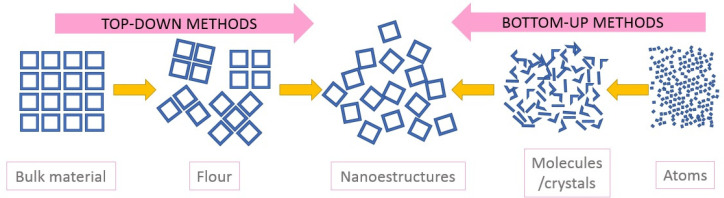
Synthesis methods of nanoparticles and nanostructures.

The nanoparticles that were used in the reviewed papers were usually provided by industrial suppliers, as in [61], either in the form of flour or a concentrated dispersion, with defined specifications in terms of the distribution of sizes, shape, or composition. This limits the researchers’ ability to control variables which affect the stability of the nanofluids, such as the size and surface characteristics. As a result, many researchers have synthesized their own nanoparticles in order to control these aspects and study their effects on nanofluid properties. The methods that were followed for this purpose are shared by various branches of nanotechnology; they can be split into two categories: bottom-up, and top-down methods, represented in Figure 1 [144].

Top-down methods involve the reduction of the particle sizes of a pre-existing bulk material by grinding, or vaporization-condensation; in bottom-up methods, nanoparticles are created by a chemical reaction or the physical interactions of precursors, atom by atom, as in coprecipitation or solvothermal techniques. Although the bottom-up methods are more complex, they are more versatile, since the rate of addition of reagents to the solution and its concentration [145,146], together with the time of reaction [21,55,66,108,146] or temperature [147], affects the mean size and composition of the nanoparticles, and can be used to control them [109,139]. By reducing the reaction time or concentration of the reagents, smaller particles are produced, although the dependence of size on time was not seen in all of the studies [23]. Others have found a relationship between the size and the moisture that is present in reagents during synthesis [148,149], which may promote the growth of particles.

Several examples of bottom-up methods were found in the reviewed papers. Starting with metal oxides, ferrous and ferric oxides arise from the co-precipitation of their cations from respective chloride, nitrate, or sulphate salts in a watery solution following the addition of bases such as ammonia or sodium hydroxide under shaking [137]. This procedure, based on pH changes, was followed in many works [107,145,149,150,151,152,153,154,155], including those specific to thermal-dielectric nanofluids with transformer oils [8,20,34,58,96,123,124,156]. Alternative methods used the reaction of iron precursors in alcoholic-organic solutions [23,55,139,146,157], and again there were examples in thermal-dielectric research [7,19,21,55,66,108,109].

These processes produce different iron oxides, classified as a function of their Fe^2+^/Fe^3+^ ratio and predominant allotropic phase, as magnetite (Fe_3_O_4_), maghemite (α-Fe_2_O_3_), or hematite (α-Fe_2_O_3_), among others. The abundance of the different phases depends on the concentration of the reactants, the temperature, and the presence of an oxidant or inert atmosphere. If magnetite is preferred over the other phases, a 2/1 mol ratio of ferric/ferrous salts is commonly used [8,20,34,137,149,150,152,154,155,156,157,158], (or even lower, [107]); nitrogen environments are also used [55,107,108,153] to offset the oxidation of ferrous cations during the reaction. In other cases, the production of maghemite was promoted by the addition of oxidants, or by bubbling a heated solution with oxygen [20,158]. Some of these particles also include cobalt [23], magnesium, manganese [124], or nickel oxides [124,159] in their composition in addition to iron oxides.

Solvothermal processes are frequently used in the synthesis of titanium and zinc oxides [101,148,160,161], they are also used to evaluate the effect on the thermal-dielectric properties of fluids [3,25,57,63,65]. During these processes, organic precursors of titanium or zinc react with organic solvents, frequently followed by calcination [65,148,160]. Again, the characteristics of the products depend on the process that is followed, e.g., which acid is used as a reagent [161].

Organic nanoparticles, such as CNT and graphene, are also very common research subjects in a wide range of disciplines, recently including nanodielectrics [116]. CNT were commonly produced by the bottom-up catalytic decomposition of methane diluted in hydrogen [142,162]. Other works used a different process to obtain graphene [116]. First, graphite flour was oxidized in three steps by sulphuric acid, acid salts, and hydrogen peroxide, to produce graphene oxide (GO). Second, the oxide was reduced by sodium borohydride or hydrazine hydrate with ammonia. Other organic nanoparticles have been produced using other techniques; for example, nanocarbon was condensed by dispersing water over the smoke from the combustion of acetylene in a leakage of oxygen [163].

Top-down methods are more frequently used for the formation of metallic nanoparticles [2,143]. Metallic wires with a diameter less than 0.25 mm are exploded by subjecting them to a voltage in a liquid medium. However, there are also examples of the bottom-up production of metallic nanoparticles, such as those using copper acetates in a hydro-alcoholic solution [164]. Similarly, there are examples of both methods for other compositions such as binary salts [73,165].

Sometimes, a combination of different methods may be used in the same synthesis process. For example, carbon nanotubes may be chemically shortened using nitric and sulphuric acids, before magnetite is produced by a reaction on the surface of the tubes, thus coating the tubes [145]. Other processes put bottom-up synthesized or commercial particles through a top-down milling step [61,73,148].

The reaction temperature varies depending on the production method and the reactions that are involved in it, ranging from the rated temperature to approximately 1000 °C. According to the literature, oxides can be synthesized by co-precipitation at room temperature [8,149,152,153,157] or at approximately 100 °C [7,19,20,34,107,123,124,137,145,151,154,155], or by the decomposition or solvothermal way at approximately 200 °C [23,101,146,159], 300 °C, [21,55,139], or even above 500 °C, [3,65,160]. Sometimes, the complete synthesis process is subdivided into several steps, each at a different temperature [7,19,21,55,66,108,109].

The solvothermal method and its variations, requires high temperatures and pressures, as illustrated in many methodological descriptions [18,25,65,101,157,160], in order to improve the solubility of reagents and to promote their reaction. The consequent calcination takes place between 450 and 500 °C [65,148,160].

Metallic nanoparticles that are created by bottom-up methods using precursor reactions need similar temperatures to metal oxides, approximately 100 °C [164]. Meanwhile, the catalytic decomposition of methane for carbon nanotubes requires a higher temperature of 1000 °C in order to take place [142,162].

Table 6 collates this classification of references, which can be used to discern which synthesis procedures are suitable for laboratory equipment as a function of temperature.

### 2.3. Nanoparticle Surface Treatments

Depending on the expected application of nanoparticles, further treatments may be necessary, where surfactantion is the most common when nanofluids are involved. This consists of the coating of particles with products that make them more prone to remain in suspension [5].

Substances are selected for this is according to the particle and base fluid [25,38,124,151], environmental conditions that they must withstand [22,142], and the effects of the surfactant on the properties of the fluid [73]. Considering the addition of nanoparticles to an oil or ester, most of examples that we reviewed show surface functionalization with organic acids of different hydrocarbon chain lengths, mainly oleic acid (55% of the papers where the presence/absence of a surfactant is mentioned). Table 7 summarizes the products that were used as nanoparticle surfactants in the reviewed papers, including the composition of the particles that were treated and the base fluid of the nanofluids that were prepared with them. The length of the organic acids regulates the particles that are susceptible to being functionalized, as shorter molecules are only suitable for dispersing a lower fraction of the particles in suspension [166].

These can be included either as reagents during the synthesis of particles [8,21,25,55,59,65,66,107,108,109,133,139,156,157], added to nanoparticles [7,19,34,51,57,58,61,66,70,73,85,91,96,113,124,137,148,149,151,152,153,158], or at both times [107]; they can be in a aqueous solution [20,25,67,107,123,124], organic solvent [7,19,21,51,59,61,73,108,109,128], or oil [34,107]. When using water as a solvent and organic acids as the surfactant, it may be convenient to add substances to enable the contact with the particles, sodium hydroxide or organic solvents such as acetone, for example [107,150].

**Figure 2:**
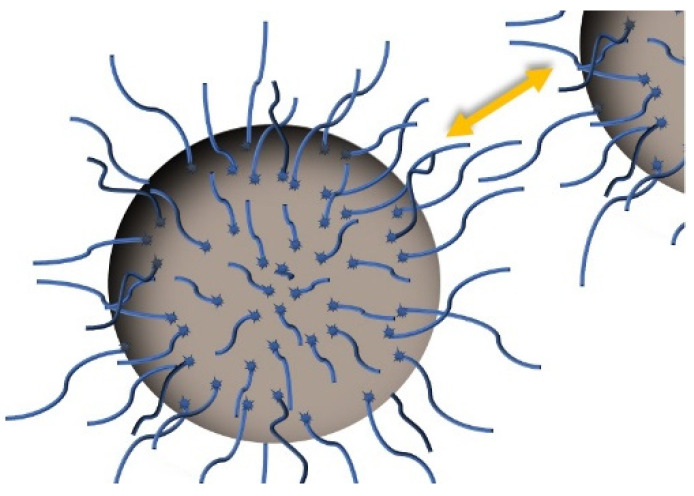
Steric repulsion between surfacted nanoparticles.

Under shacking and with the proper amount of energy, acid molecules are adsorbed by particles through physical or chemical interactions; since the latter are stronger, they are better at maintaining the coating [20]. Theoretically, the carboxylic groups in surfactant molecules bond to the nanoparticle surface in different ways [151]: keeping organic chains towards the surrounding medium, changing surface polarity [64,75,149], and occupying the active surface [110]. Due to their length, these chains hinder approaching particles by increasing the osmotic pressure, which is known as steric repulsion and is represented in Figure 2. The larger distances between the particles means that the strength of the van der Waals forces and polar interactions among them are reduced [23,84,90,110,139,167]. Additionally, interactions between these chains and the surrounding oil are enhanced as organic acids are soluble in it [27,61,130,164]. Therefore, this creates coated particles with both an improved resistance to aggregation and relationship with the surrounding oil. This theory is demonstrated in some works by the fact that these particles tend to migrate from water to the organic phase when they are put together [20,158], and that the mean size in suspension is much lower when a surfactant is used [23,30,101], A beneficial side effect is that the coated particles, which were susceptible to oxidation, are protected [34,40,107,164].

Treatment is often carried out above the rated temperature in order to assure the adsorption of these reagents [7,8,19,58,66,73,96,108,109,123,124,129,156]. In the cases that we reviewed related to dielectric nanofluids, the temperatures during surfactantion were approximately 80–90 °C [8,34,73,129]. Another important factor is the surfactant concentration. As shown in Table 8 (in grams of surfactant per liter of fluid, volume percentage, milliliters of surfactant per gram of nanoparticle or weight percentage), the quantities of surfactant that were used during treatment were relatively low; it must be below the critical micelle concentration to avoid its own aggregation and negative consequences for fluid viscosity [168]. An optimal concentration from the point of view of stability or nanoparticle size distribution can be achieved [30]. In some examples, the surfactantion process was repeated to increase the surfactant adsorption [21,107].

Surfactants maintain the crystal structure of the treated particles [23,148] and slightly increase their size [23]; on the other hand, modifications of the pre-existing surface of particles also have been seen by the addition of acid substances [34,137,162].

### 2.4. Nanoparticle Recovery from Dispersion

Once the particles are ready, after synthesis or surfactantion, they must be recovered from the reaction solution. A number of methods were used in the papers that we reviewed, including: filtration [61,65,124,148], centrifugation [20,21,51,55,65,66,101,108,109,116,124,139,148,149,151,152,157,159,161,164], heating [128], and natural [158] or magnetic decantation [8,23,34,107,123,124,137,150,153,155,156,169] for magnetic particles. Sometimes chemical agents were added to reduce the solubility of the particles in the solvent in order to enable their extraction [21,66].

This step can be used to select specific particles. For example, during natural decantation, it is possible to take just the particles in the organic phase and to return those in the aqueous phase to the previous processes [20,158].

**Figure 3:**
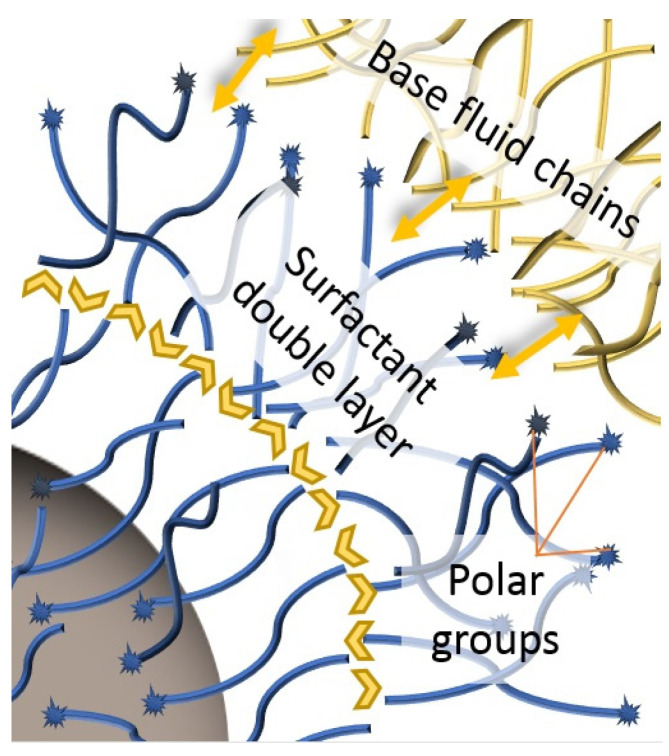
Hydrophilic double layer of surfactants and dielectric fluid.

Recovery is followed by different phases of washing to eliminate excess reagents and solvents, or to optimize the surfactant coating [55,150,151]. During the formation of the surfactant coating, carboxylic groups bond chemically with the particle surface; this makes them lipophilic due to the hydrocarbon chains. However, if the saturation amount of surfactant is exceeded, these extra molecules interact physically with those that are attached to particles through their chains, creating a second layer [61,90,107,110,148,170], represented in Figure 3. For carboxylic groups facing the fluid [61,110,148,170], this gives the particles a polar or hydrophilic nature which, although not suitable for addition to and sustenance in transformer oils, is sought for certain applications [107,170].

The necessary amount of surfactant for each situation is difficult to know as it depends on the composition, concentration, morphology, and the size of the particles, so washing is a suitable way to remove the extra layer. Water [34,107,116,137,149,152,153,154,155], alcohol [65,139], organic solvents [23,123], or a combination of them [7,19,20,25,101,108,109,146,148,150,151,157,159,164,169] are used in this step. After mixing and shaking, the nanoparticle must be recovered again. To eliminate the remnants from washing, or moisture in general, the particles are dried by heating them at temperatures up to 100 °C, in air or a vacuum, for between 12 and 72 h [7,8,19,20,25,34,51,61,66,101,108,109,137,139,146,148,151,152,156,158,159,161]. Alternatively, a nitrogen gas stream can be used for the same process [20]. The drying processes can affect the size distribution of nanoparticle flour since they promote aggregation [73]. Particles are thereafter kept in the form of flour or suspended as a concentrated nanofluid.

### 2.5. Preparation of Nanofluids

**Figure 4:**
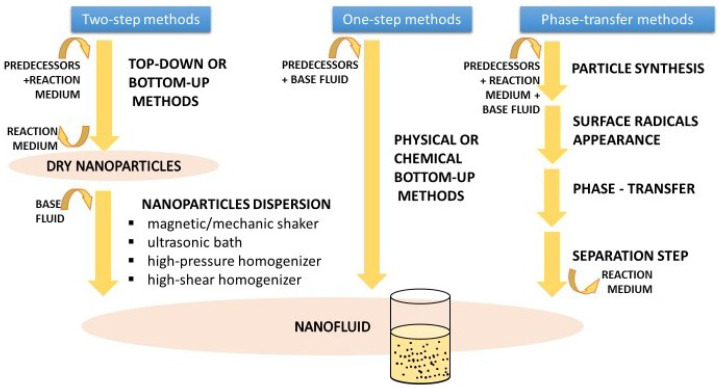
Available preparation methodologies for nanofluids.

As one of the main branches of nanotechnology with several potential applications in different fields such as medicine and engineering, different nanofluid preparation methods have been developed and adapted for their final characteristics by researchers and producers based on the raw materials and available equipment. A compendium is available describing the wide range of production methods [5]. This explains that they are classified in three categories: two-step and one-step methods, and all other methods. The first two differ from each other in how nanoparticles are introduced into the base fluid. Two-step methods first produce nanoparticles as dry powders, which are added and dispersed in a second stage, while one-step methods produce nanoparticles directly within the final fluid. Other methods require the transference of nanoparticles between phases during synthesis; this takes place when new radicals appear on the surface of the particles, changing the inclination of solids to a determinate fluid. These alternatives are represented in Figure 4.

Every methodology presents its own disadvantages [5]. In two-step methods, as particles must be recovered from the reaction solution and kept as a powder, aggregation processes take place. Dispersion in the base fluid, which is carried out by magnetic/mechanic shakers, ultrasonic baths, or homogenizers that are used to ensure a proper distribution of particles, are useful in breaking up the aggregates created during handling. Despite the dispersion steps, aggregates continue to be created. This may have consequences for the stability of the nanofluids as aggregates migrate to the bottom of the dispersion faster. Therefore, surface treatments with surfactants to avoid aggregation become necessary. The main problem with one-step methods is that, as the nanoparticles remain in reaction fluid, so do the reaction waste products and the residual reactants; these are able to distort the nanoparticle effects over the base fluid properties [5]. Moreover, there is little knowledge about the characteristics of these additional particles.

Two-step methods were followed in most of the papers that we reviewed. Regardless of their origin, a certain amount of nanoparticles were added to a nanofluid preparation until the desired concentration in the base fluid was achieved. One-step methods were found only in articles using water [171,172]. Thermal-dielectric nanofluids are habitually based on mineral oils, but there are several examples being prepared using esters. In this case, natural esters prevailed over synthetic alternatives, as shown in Table 9. Frequently, base fluids suffer pre-treatments such as filtration or drying to eliminate impurities that affect their performance as an isolation medium, as specified in the regulations for transformer oils by the International Council on Large Electronic Systems (CIGRE).

To disperse particles in the base fluid, in these cases both magnetic/mechanical shakers and ultrasonic baths are used [7,8,19,25,50,65,66,73,85,92,95,107,108,109,150,156], frequently for less than 30 min, especially with the former, according to Table 10. The aggregates are thus broken apart, ensuring the homogeneity of the nanofluids. Therefore, sonication is sometimes used as a final step in the particle preparation as it occurs with milling [61]. An alternative method of homogenization is available where a mixture of the base fluid and solid fraction are passed through a high-pressure homogenizer that divides the sample stream into different micro-channels [26]. The mixture suffers high velocities, shear forces, crashes, and cavitation, which break the aggregates apart and disperse the particles. Another method uses a high shear homogenizer that breaks the aggregates apart by friction, with samples set between its stator and turning rotor [179].

During these phases, especially with ultrasounds, the samples are exposed to heating [184] which can harm the bonds between the surfactant and particles. In some articles, variations on the general method of dispersion are used. For example, the dispersion process may be repeated several times to eliminate excess surfactant completely, in an attempt to optimize the coating of particles and to avoid the formation of a double layer of organic acids [123]. In other studies, sonication is implemented with regular stops to prevent the excessive heating of samples under treatment [25,68,163,174], while others include a cooling system [184].

Once the nanofluid is prepared, before its application, it is frequently dried under heat or/and vacuum, as with nanoparticles, for at least for 12 h [18,24,53,62,73,85,92,95,108,109,115,133]. This enhances the dielectric strength of the fluid, prevents the formation of new bubbles, gives the fluid time to dissipate existing bubbles [3,7,19,63,88], and can even eliminate traces of volatile solvents [61].

### 2.6. Nanofluid Stability Assessment

**Figure 5:**
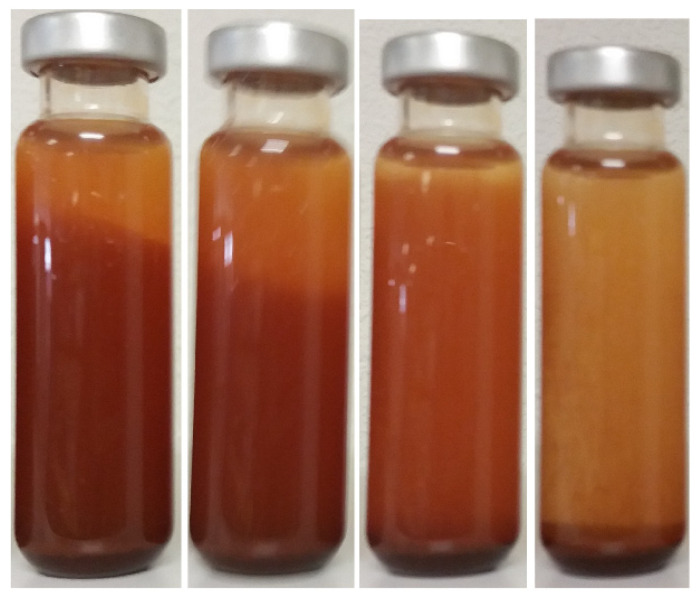
Oil-based nanofluid dispersion evolution with time.

The stability of nanofluids can be measured first by sight [12,18,20,21,60,66,110,132,162,179], as when sedimentation of the solid fraction occurs, the appearance of the suspension changes. An initially homogenous suspension will develop different zones: the upper zone is similar to the base fluid, the intermediate zone is opaquer and it is colored by the nanoparticles, and the lower zone is composed of settled wet particles, as shown in Figure 5. The widths of the zones evolve with time; as the first and last grow, the second decreases until it vanishes completely, which takes longer in more stable dispersions. Some studies accelerate this process by submitting the samples to centrifugation, which is also faster as the stability decreases [5].

To achieve accurate and objective measurements better than human sight, there are techniques to assess the stability of nanofluids, such as colorimetry and turbidimetry, which compare the different stages over time. Alternatively, the evolution of the mean size of dispersion, measured using dynamic light scattering (DLS), can also be useful as this is the parameter that is first-affected by aggregation [59,70,86,90,106,110]. Stability can be assessed based on the magnetic field that is used to separate particles from the dispersion [107] or by controlling the concentration evolution of the dispersion [27].

Other techniques are based in properties that are indirectly related to stability, as Z potential measurements. The Z potential reflects the difference in potential between the dispersion medium and the layer of fluid that is closest to the particles, typically a double layer of ordered molecules. The larger the Z potential, the more electrically stabilized the suspension [5,115], according to Table 11 [6]. Additionally, the electrostatic repulsion between the particles increases with the surface charge of the particles in the fluid [27,89,108]. When the base fluid is water, it seems reasonable that the stability of the particle suspension will increase as the electrostatic interactions grow, as is widely described in the literature [22,112,163,170,179], where the stability of a nanofluid is defined as a function of the Z potential magnitude.

In the case of nanofluids that are based on oils, contradictions have been found. It was mentioned earlier that in this case, the optimal coating of particles with surfactant is believed to be a monolayer, but this supposes that organic non-polar chains are on the surface. According to the definition of the Z potential, this situation corresponds to the zero potential; this is supported in the literature [170], but the opinion is not shared by other researchers who link the monolayer and the resulting stability to the higher absolute value of the Z potential [72,89,115,124,143]. In the same way, they disagree on what tendency the Z potential shows during the formation of a second layer of surfactant. One fact that supports the most common interpretation is that in one reference the potential was higher at higher temperatures, and thus more stable [143]. Similarly, in other studies the optimal properties were found in those samples whose Z potential was higher [21,25].

Unlike Z potential measurements, UV spectrophotometry is not constrained in viscous [27] or concentrated nanofluids (over 0.1% volume [185]). It is based on the change in the light absorbance of nanofluid samples over time. As with sedimentation, where the concentration of the particles and turbidity decrease over time, so does the light absorbance. Some examples of the application of UV spectrophotometry in oil nanofluids were found in the papers that we reviewed [27,29,94,101,129].

The specific test for magnetically-induced aggregation is acoustic spectroscopy. The attenuation of an acoustic wave depends on the structure of the material that it propagates through, showing very different and defining results if the wave propagation and linear-aggregate directions match [33,167].

Before preparing nanofluids, in order to check if nanoparticles are suitable for transformer oil, the samples can be dispersed in water/oil mixtures. They are suitable if they remain inside the organic phase [151]. Moreover, to quantify this tendency, a lipophilic grade can be measured; this is the amount of alcohol that must be added to the mixture to cause the particles to migrate to the watery phase [148].

The stability gaps that were shown by the oil-based nanofluids that were prepared by the authors of the different references, according to themselves, are given in Table 12. These are the time that lapsed from the preparation of a nanofluid until its solid content is clearly at the bottom of the vessel, following the evolution that is seen in Figure 5. Conclusions can be drawn from this information. For the studies that noticed a life span of more than one year, and for which information was available, a number of techniques and design choices were applied during the preparation of the nanofluids. This included low concentrations [20,21,25], small mean sizes [20,21,25,41], optimized mixings [25], and lipophilic particles (naturally [12,29] or by treatment [20,21,25]). In the last case, particles were submitted to washing [20,21,25] or preselection processes [20], and the surfactantion was boosted thermally [21,25] or chemically [20]. It must be noticed that the life span of the dispersions that are based on natural esters (more viscous than the mineral alternatives) were over 1 month, and 55% of the samples which were stable for more than one year were titania and mineral oil nanofluids.

On the other hand, unstable nanofluids have been produced with higher mean particle sizes [82,87,88,110], higher concentrations [87,143,156], and without the surface treatments of particles to make them more lipophilic [64,82,130]. Only two of the nanofluids whose surfactantion was thermally promoted were classified as unstable [8,156].

Comparing both situations, it seems that only those nanofluids that combine the proper size, concentration, and surface conditions may last the required time.

## 3. Achievements Found in Literature

Although convection is the main mechanism of heat transport when fluids are the cooling medium, nanoparticles are included in the base fluids with the purpose of improving the contribution of thermal conduction. The thermal conductivity of solids is several times higher than that of fluids, by one or two orders of magnitude [88], so theoretically the thermal conductivity of a nanofluid would be intermediate between those of its components, an increase of as much as the solid fraction in suspension. Also, because the convective coefficient depends on the thermal conductivity of the cooling fluid [186], both may be enhanced by the presence of nanoparticles. Nevertheless, this presence could be detrimental to convection, which depends on the nanofluid’s viscosity and evolution with temperature, but this parameter is affected by the nanoparticles. Additionally, improvements in conduction are constrained by the concentration limits that are set by the stability requirements. Again, a compromise solution between all of these parameters must be achieved.

Thus, since the proposal of cooling nanofluids, researchers have focused on demonstrating these tendencies, and have tried to determine how to achieve a meaningful improvement in conduction without harming convection, or any other essential properties of the base fluid.

### 3.1. Cooling Capacity

Several direct measurements of the thermal conductivity of nanofluids have been done with samples based on a variety of cooling oils. The aim was not only to verify the effect of nanoparticles on conductivity [25], but also to show how different variables affect it. Logically, one of the variables was the concentration of solids, but others such as nanoparticle type, environmental conditions, and preparation method were also explored. These variations let researchers identify greater increases in conductivity, giving an idea of the potential improvement that nanoparticles could bring. These results are shown in Table 13, where it can be noticed that although there are examples where the variation was negative or below the confidence interval (5–10%), most of results show maximum increases of more than 20%, and some are approximately 100%. Some mathematical models support increases in the conductivity of oils by more than 500% [8,126]. To help with the comprehension of these results, Table 14 summarizes some characteristics of these nanofluids from previous sections of this review.

Thus, several of the papers that we reviewed support the theory that thermal conductivity grows in line with the nanoparticle concentration [8,21,26,39,61,73,88,115,124,129,130,131], although there is not unanimity [64]. This trend is supported when the results are from different sources, which use nanofluids that share most characteristics except the concentration of solids, are compared, as with AlN in [61,73].

Together, the research points to an optimal preparation concentration, as the growth of conductivity decelerates at higher concentrations [73,124]. Other studies show that the growth in the conductivity is not linear before saturation, but it grows at an increasing rate [130].

The variety of nanoparticles or the pre-treatments they suffer, can condition these results. It has been shown that the conductivity variations of nanofluids with a similar concentration can differ due to changes in the particles’ characteristics, such as their composition [26,61,91,131], allotropic structures [25], or shape [26,61,130]. Some effects can be explained by the conductivity of the nanoparticles’ bulk material [26,91,113,115,131]; nevertheless, the lack of specific investigations comparing their effect on the function of the composition prevents us from making conclusions about which of them are better. Changes in the conductivity may also be a result of differences in the nanoparticle size. In addition, when considering surfacted particles, it has been noticed that the surfactants themselves can increase the thermal conductivity of the base fluid; the higher the concentration, the bigger the increase [61,73]. However, this effect is small compared to the nanoparticle effect on the base fluid [58,73].

Environmental effects on the thermal conductivity of nanofluids were also addressed. The natural tendency of the thermal conductivity of oils is to decrease as the temperature increases [21,131,143]. This was observed in some of the investigations with dielectric nanofluids [64,91,113,124,131], but the opposite behavior was seen in others [21,39,130], as it was in some oils [22,143]. Considering the magnitude of the conductivity variation with temperature, larger relative increases were observed in the base fluid at high temperatures [21,129,130], with signs indicating the existence of an optimal temperature [21]. Nevertheless, other studies have shown linear variations, as it is theoretically predicted [91,124,131].

Magnetic fields positively affect the conductivity of nanofluids for a determined concentration, and with a saturation tendency at higher fields [8]. The magnitude of this enhancement depends on the directions of the field and temperature gradients and, when they match each other, it is maximal. It seems that particles are aligned according to the field direction, creating a preferential direction for heat transport by conduction, which is able to provide a five-fold increase to the enhancements [8].

**Figure 6:**
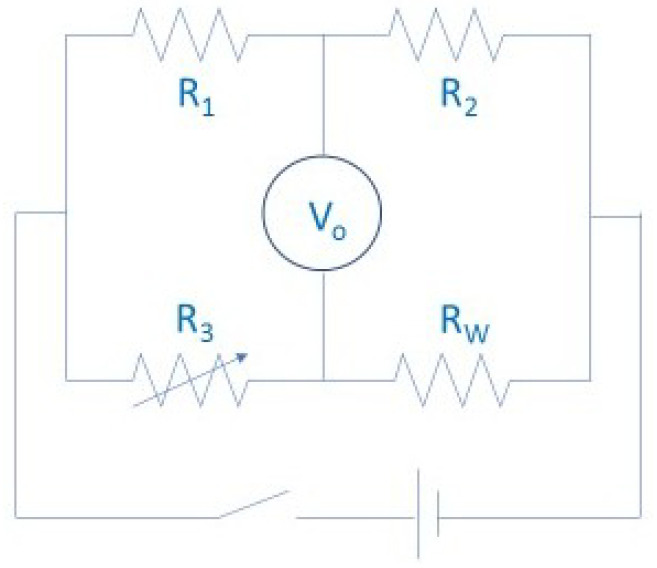
Hot transient wire scheme.

These measurements were carried out with accurate equipment, which was designed for conductivity analysis in other materials, mainly solids, but was adapted to fluid requirements. During the experiments, convection was generally limited in the samples by fast measurements and constrained fluxes of oil. These were largely based on hot transient wires and discs [8,22,26,27,49,61,64,73,88,124,131,143], represented in Figure 6, which were heated by an electrical heat source at the same time as a probe. The thermal conductivity results were found by linking the changes in resistivity (R_3_), voltage (V_0_), and temperature in the wires due to the heat dissipation across the tested material. The temperature is controlled by measuring the electrical parameters of the circuit, and the thermal conductivity of the probe surrounding material can be obtained by comparing the evolution of the temperature and the amount of heat that is produced by the electrical circuit. Other experiments used the temperature oscillation technique [175], 3ω-wire method [165], or laser flash method [25].

According to the literature, natural convection [49,123] and overall heat transfer [61,110,118] are also enhanced by nanoparticles, with similar results seen for conductivity in terms of the concentration of nanoparticles [61,118] and surfactants [110]. More precisely, the improvement in conductivity was lower than that which was found for the overall heat transfer coefficient, pointing to an improvement in convection [61]. In the same way, the shape and composition of nanoparticles may also affect the heat transfer [61].

Characteristics that could condition the convection performance of a coolant oil, such as viscosity, especially with natural flux, were not generally harmed by the presence of nanoparticles. Although slight increases were found [49,51,56,58,61,81,86,91,94,111,115,127,180], they were just as frequent as those with no effects [12,25,26,51,59,60,62,64,98,119,121,123,130]. Indeed, reductions in the viscosity were also noticed [22,51,84,91,95]. Researchers have demonstrated that the resulting viscosity depends on the nanoparticle concentration [26,51,56,58,111,115,126,127,130]. To ensure stability, nanofluids were prepared with low concentrations, and the particles were mainly spherical; it is reasonable that the viscosity was not significantly affected as a result. Another idea is that nanoparticles may act as a lubricant between the layers of fluid, as they remain oriented in the flux direction [26]. The viscosity of nanofluids decreases as the temperature increases, such as with the base fluids [49,51,56,58,59,64,86,94,123,127], and seems to be enhanced by the presence of external electric fields, as a function of the nanoparticle concentration [71,119]. Nevertheless, an essential modification was identified, which changed the behavior of the base fluid from a Newtonian to a non-Newtonian fluid at a determinate concentration of nanoparticles [22,26,143] or at low temperatures [143], due to an increase in the number of interactions [130].

Heating tests with nanofluids samples were also done, controlling the temperatures in the fluid, isolators, and heaters. Here, nanofluids showed better cooling capacities and the temperatures that were measured in the tester components were lower [49,69,76,77,80,118,119,131] and those in the coolant were higher [21,80,129] than in tests that were carried out with the base fluids, with some exceptions [60]; the time that was required to reach these temperatures was also lower [21]. It was noticed that these differences became more pronounced at higher particle concentrations [21,80,119,129] when the base fluid was less thermally conductive [131] or when an external electric field is applied on the nanofluid cooling circuits [119]. The existence of optimal concentrations has also been noticed [118].

Similar results were obtained in other studies with other base fluids such as water or ethylene-glycol, which showed improved conductivity [172,173,175,176,179,183] and convection coefficients [173] as the concentration of particles increased. These results were dependent on the composition [27,175], shape [142,183], and size [173,176] of the nanoparticles.

**Figure 7:**
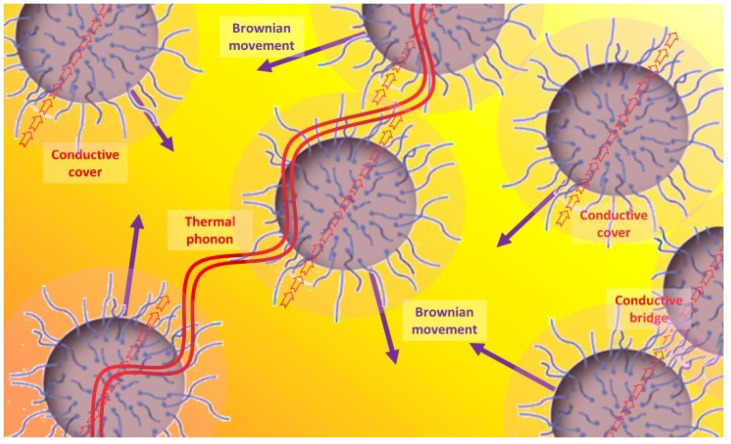
Heat transference mechanisms in nanofluids.

Explanations for these phenomena were given according to four main theories, represented in Figure 7 [49]. These are the Brownian movement of nanoparticles in fluids that are caused by the heat (purple arrows), particle-fluid interface behavior (darker corona around the particles and red arrows), ballistic phonon transport (red waves), and higher thermal conductivity of temporary particle clusters (red arrows shared by two different particles) [129,187], as mentioned earlier.

The first explanation justifies the improvements in the convection and its coefficient because of the Brownian movement of nanoparticles in the base fluid [16,22,39,87,143,165,171,175,176,178], especially for spherical [165] and small [176] particles. This movement would be linked to the fourth explanation. According to this, the nanoparticles continuously form temporary clusters due to their movement, that create conductive paths for thermal transport [27,39,73,87,130,165,175,177,181]. Considering the second explanation, the molecules in the layers of fluid that are closest to the particles apparently tend to keep more regular organization, resulting in behavior that is more similar to that of solids and a higher thermal conductivity [130,131,177,178]. Finally, according to the third explanation, thermal phonons are able to have ballistic transport between close nanoparticles due to their wavelength and the distances and diameters that are involved [16,130,131,177,178].

Increases in the dispersion concentration or temperature would enhance the magnitude of Brownian motion and temporary clustering [130], and can explain the behavior that was observed with these parameters, when caution is taken not to promote settlement. The differences that were seen for different particle compositions may be due to their bulk material conductivities [26,131], or because one kind of particle is more prone to maintain stability than others in specific circumstances [22].

Another property which has been studied is specific heat; it has been addressed by a few researchers using dielectric oils. These results demonstrated that the specific heat decreased when nanoparticles were added [69,131], and the effect was more pronounced as the concentration of particles increased [124,131] (as in watery nanofluids [163]), or when their thermal conductivity was bigger [131]. The specific heat also tended to increase with increasing temperature [69,124].

### 3.2. Flash, Fire and Pour Points

From a thermal point of view, not only the cooling capacities of nanofluids are important. If the aim is their use of equipment under high working temperatures, such as transformers, security must be considered. Dielectric oils and the gases they create, are potentially flammable, so the effect of nanoparticles on flash and fire points must be controlled. Again, not many investigations were found in this direction, but most results seem promising as these temperatures rise slightly due to the presence of nanoparticles [51,81,94,121], not more than the 3 °C seen with other kinds of base fluids [181]. In the worst case [111], a decrease of 8 °C (5.12%) was noticed.

Regarding the effect of nanoparticles on oil pour point, depending on the nanoparticle, it can be increased or decreased [51].

## 4. Dielectric Properties

In light of the application of nanofluids in power transformer cooling, the need to characterize their dielectric properties arose naturally. Dielectric cooling fluids must be able to withstand voltages that are greater than the rated values of the equipment in order to ensure the operation and safety of the components in case of an electrical fault.

This capacity was expected to decrease in dielectric nanofluids in comparison to the base fluids, due to the presence of nanoparticles, as occurs when the transformer oils are not prefiltered in line with the recommended standards [3,42] or with microparticles [41,94]. Nanoparticles are often more electrically conductive than transformer oils, so theoretically, this may harm the fluid’s ability to hinder or delay discharges and streamers. However, the results were unexpectedly good, driving interest in this field of nanotechnology. This improvement in the dielectric properties was observed using many of the variety of tests that are available.

### 4.1. Dielectric Strength

**Figure 8:**
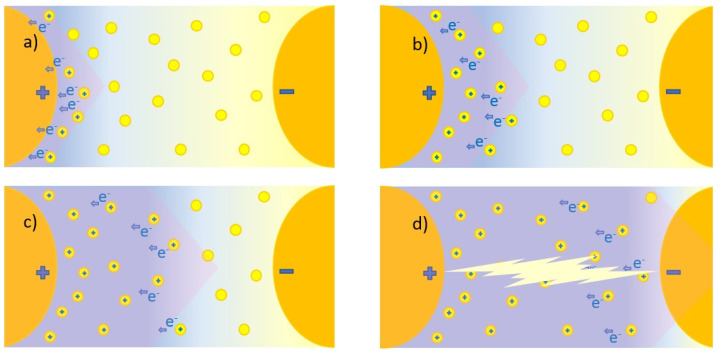
Streamer development and discharge in dielectric fluids: (**a**) Creation of ionized area in the positive electrode by migration of the electrons, (**b**) Increasement of the electric field in this area that promotes more ionization and migration of the electrons, (**c**) propagation of the ionization towards the negative electrode, (**d**) Completion of the ionization between the electrodes and occurrence of the discharge.

The dielectric strength represents the dielectric fluid capacity of withstanding voltages without the formation of a conductive channel through which an electric current may be established. This capacity is not constant; it depends on the magnitude of the electric field, as it might be sufficient to promote the ionization of fluid molecules and subsequent warming and gas formation, which finally translates to the enhancement of the electric conductivity, represented in Figure 8. Here, there are represented the electrodes that creates an electric field. The yellow circles represent the molecules inside the base fluid, polarized by the field. During ionization, slow cations and fast electrons are created from the oil molecules (a); the electrons tent to migrate towards the anode or positive pole, while the cations create a positively charged area. If the required conditions are fulfilled, this could enhance the local electric field, promoting more ionization in the surroundings and providing feedback to this process (b). This results in the conductive red zone spreading in the fluid, an effect that is known as streamers (c). When completed, a conductive channel of ions and gas is available, and discharge takes place (d). This process, mentioned in several references [10,28,38,67,83,84,89,120,134,136], is supported by simulations [188,189] in which an ionizing wave was observed by representing the estimated electric field, temperature, and the electric net charge over time. Discharge occurs from a determined voltage, dependent on the dielectric media, known as breakdown voltage (BDV).

Studies of the BDV have been developed by a narrow range of tests, to be representative of all the possible types of dielectric fault. This includes direct (DC) or alternating current (AC) breakdown voltage tests, where the samples of dielectric nanofluids are submitted to increasing voltages inside a cell until an electric arc appears between two electrodes, according to IEC 60156 standards for a lapse of time. Lightning impulse breakdown voltage tests are also common (IEC 60897), with the difference that, while the stresses in DC or AC breakdown tests are less acute, lightning impulse tests involve the application of higher voltages for shorter time intervals [14,37,40,83], thus including this kind of failure in investigations. Finally, partial discharges are analyzed (IEC 60270) by finding the voltage that is necessary for their appearance, called the partial discharge inception voltage (PDIV), where localized currents reach a determined magnitude as a function of the background noise [43,44]. Once the PDIV is known, tests are carried out at or above this voltage, measuring the magnitude and concurrency of the discharges [11,28,62].

The following tables collect the results from these tests that were carried out under different conditions. Starting with those from alternating current tests, Table 15 shows the range of improvements that were reached in each of the studies that we reviewed. Dispersion in the conditions of nanofluids makes it difficult to notice if a specific component is more prone to greater increases in the breakdown voltages, although they seem more constrained in vegetal base fluids, as their starting BDVs are higher. It must be remembered that the breakdown voltages of the base fluids were already above the required values as they are applied in actual transformers, so any increase would be a step towards more reliable and powerful equipment. Although not all of the tests reflected improvements, most of them were more than 10%, higher than the estimated error of the measurement techniques.

**Figure 9:**
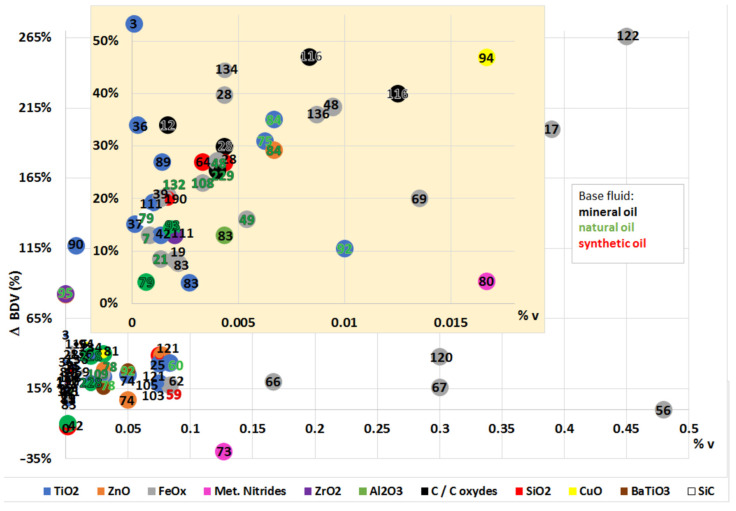
Comparative of the BDV (AC or lightning impulse) maximal variations depending on the base fluid and the volumetric concentration and type of nanoparticles.

In the Figure 9 the different maximal breakdown voltage variations that were noticed in the reviewed works are presented, including the information about the composition of the nanoparticles that were used during the research (represented by the color of the spot in the graph, explained in the legend), their concentration (volume percentage), and the type of base fluid (depending on the colors of the bibliographic reference number, black for mineral oils, green for natural esters, and red for synthetic esters). A deeper view is possible with this figure as it is easy to notice the range of variations that are usually reached with the addition of nanoparticles, as well as which are the most common in these kinds of work, and at which the concentrations or the predominance of works were carried out with mineral oils as bases.

This appears more clearly when the results are refined by statistical methods. By adjusting the obtained data to statistic distributions (frequently Weibull [12,21,35,37,40,64,79,89,93,98,99,103,116], but not exclusively [79,84]), values for the low probability cases may be inferred. These, also in Table 15, represent the minimum voltages at which breakdown could take place, so they are very useful during the design phases, as they represent the maximum voltage that the dielectric can withstand without the risk of failure. Thus, equipment is designed with assigned values that are below these limits. When comparing these statistics from the base fluids with nanofluids, it can be seen that the improvements in the breakdown voltage are frequently even better, with the majority over 20%. Less frequently, DC breakdown voltage tests have been also developed that also show enhanced dielectric strength when in nanofluids compared to the base fluids [14,16,18,45,51,52,104,131], with some exceptions [52].

**Figure 10:**
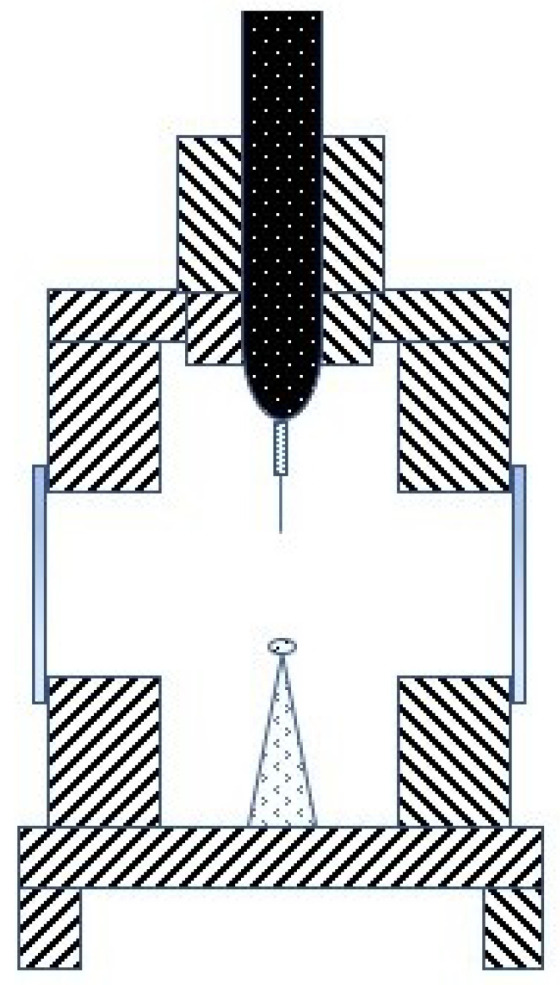
Lightning impulse tests electrodes configuration.

Similar results are collected in Table 16 and Table 17 relative to the lightning impulse and partial discharges tests, respectively. The lightning impulse results can be separated into two groups based on their polarity. Standards require that these tests must be done using a needle/sphere configuration of electrodes as shown in Figure 10 ([133]), so these tests are driven by changing their polarity, and what conditions fault development occurs is due to their different geometry.

Thus, the results are named according to the polarity of the needle electrode. The negative impulse tests have been classified together with the positive ones, but the sign is opposite. One can see increases in both the voltages and times that are required for a positive streamer to appear; this means that dielectric nanofluids are not only able to withstand higher stresses, but they also take more time to develop failures, giving extra time to stop them. On the other hand, negative streamers show decreases in the voltages and times in most of the references.

This behavior, on the whole, is satisfactory. Firstly, it is usual that the lightning impulse voltages in base fluids are bigger when the polarity is negative rather than positive. Secondly, variations in the negative voltages with nanoparticles are softer than the positive ones, as can be seen in Table 16. Therefore, the use of nanoparticles provides an opportunity to improve the lower lightning impulse breakdown voltage in this kind of dielectric fluid, while the higher voltage decreases much less, or can even improve. Similar trends are observed with the times to streamer formation.

Partial discharges seem constrained in oil nanofluids, as their PDIVs rise while their magnitude and concurrency decline, according to results in Table 17, where the percentages for the partial discharge magnitude are negative. Nevertheless, here again, not all the results were positive [11,28]; these cases show that from a determined voltage, nanofluids changed their behavior and their partial discharges went from being smaller than to larger than the base fluid in the same conditions. Also, for a voltage that is higher than the PDIV, higher acceleration of the streamer velocity was observed in nanofluids, such that they reach a fast-streamer speed at a lower voltage than the respective base fluids [40].

**Figure 11:**
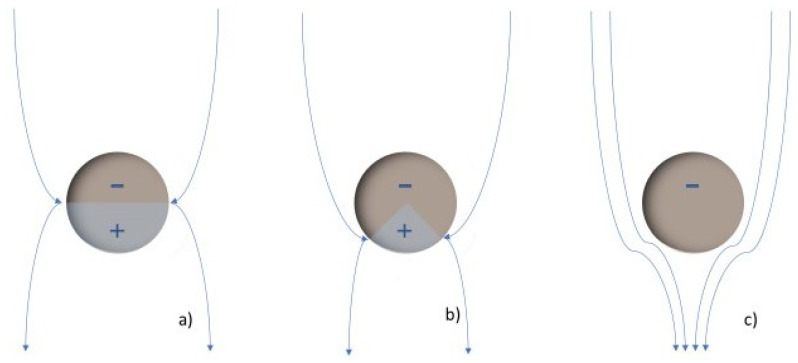
Polarization-charging of a nanoparticle under electric stress: (**a**) Distribution of surface charges in the nanoparticle once polarized, (**b**) increasement of the negative charge due to the capture of free electrons in surface potential wells, (**c**) saturation of the potential wells and the nanoparticle.

**Figure 12:**
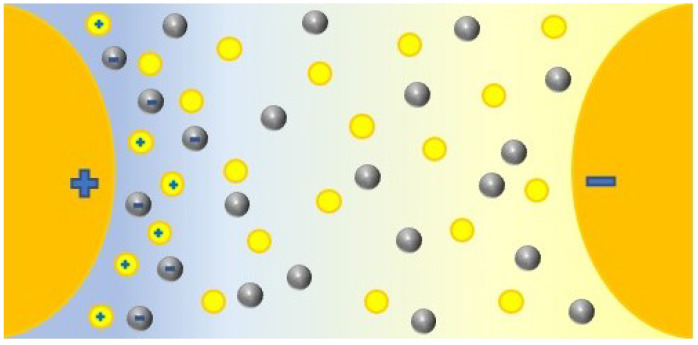
Capture of electrons by nanoparticles and delay of the streamer.

There is a spread theory that tries to explain this behavior of dielectric nanofluids based on the supposed capacity of the nanoparticles to capture electrons in their surface [3,10,11,12,13,14,17,21,28,35,37,38,39,40,41,42,45,46,47,48,52,66,68,73,74,78,83,84,98,99,100,103,104,108,111,114,116,120,122,126,135,136,188,189,190]. This may come from the polarization of nanoparticles with free charges under electric fields; this creates potential wells on the surface and leads field lines, and, therefore, electrons to nanoparticles, which are captured until saturation, as shown in Figure 11. First, the nanoparticle is polarized (a); secondly, it traps negative charges in its surface, increasing the negative charged volume, which distorts the electric field (b); finally the surface is saturated of negative charges (c). In fact, an equilibrium between the captures and liberations may be reached [12,116,188], which in practice, together with the lower mobility of particles [115], would cause a delay in the migration of electrons and their effects, hindering the appearance of streamers, regarding the behavior of the base fluid that is represented in Figure 8. Thus, in Figure 12 the role of the nanoparticles (grey spheres) is represented, that kept the free electrons launched by the base fluid molecules (yellow circles) and reduce the evolution of the electric field.

Theoretically, this capacity may depend on the particles’ easiness of polarization and depth of traps. These again depend on the particle composition, specifically on their conductivity and permittivity, and their size, respectively. In the first place, traps should be available before electron emergence as soon as possible, so polarization time, represented by the relaxation time constant (*τ_r_*), might be under streamer spreading time, in the order of nano to microseconds:

$$\tau_{r}=\frac{2\varepsilon_{1}+\varepsilon_{2}}{2\sigma_{1}+\sigma_{2}}$$

According to the previous equation [10,98], particles with a higher conductivity (*σ*_2_) and reduced permittivity (*ε*_2_) would be more suitable for application in dielectric base fluids, whose properties (*σ*_1_ and *ε*_1_) also affect the constant. In the second place, the deeper the wells, the harder it would be to scape for electrons and the bigger their capacity, thus being related to the nanoparticle sizes, yet bigger particles present deeper wells [10]. It could also be beneficial from the point of view of saturation charge of each nanoparticle [10], as their surface increases with size, although on the whole, the total available surface of nanoparticles would be minor.

Nevertheless, the particles that were included in dielectric fluids, collected in Table 5, were not only conductive but also semi-conductive or insulating, also presenting improvements of the dielectric strength that were even higher than those that were seen in similar conditions for conductive nanoparticles] [10,35,37,51,83,121,126]. It could be because polarization of these other kinds of particles takes place anyway, not with free charges, but by means of their own surface charges [10,79,83]. This theory is supported by the dielectric constant enhancement that is seen in reviewed articles and explained latterly, as it represents polarization ability.

Other sources link the capture capacity to the formation of a Stern double layer around the particles that may perform as a capacitor [37,41,42,103]. Whatever the cause of electron trapping, its effects on the dielectric properties are clear, being a plausible explanation for the trends that were found in the test results. In AC and positive lightning impulse tests, the lower mobility of the captured electrons should translates to a reduction in the local electric field enhancement at the anode, and thus the need for higher voltages and more time for streamers [17,21,47,66,74,83,99,104,126]. In negative lightning impulse tests, when the needle (where field is more accentuated) acts as a cathode, negatively-charged particles may contribute to the enhancement of the field, reducing the dielectric strength [80,83], as was noticed in the papers that we reviewed. Partial discharges nourish themselves from electrons so nanoparticles hinder their occurrence; hence, higher voltages become necessary [28,73] and discharges are less frequent and lower [11].

To try and verify this theory, specific tests were done using measurements of the energy levels and trap density. These were the thermally stimulated current (TSC) [73,104,105,106,109,114] and pulse electroacoustic tests (PEA [44,105,106,108,114]. The first involves subjecting nanofluid samples to progressively increasing temperatures after they are stressed in a continuous electric field and cooled. These samples, placed in electric circuits as if they were capacitors, will release the electrons that are caught in potential traps as the temperature rises while the electric current is controlled. The results show that currents from nanofluids are much bigger than those from base fluids, what can be read as a confirmation of the presence of a larger number of traps on the nanoparticles’ surface, although they are also present in the base fluid [73,104,105,109,114]. Meanwhile, the second test measures the time evolution of the samples which have been stressed by DC voltage, by using acoustic pressure waves that interact with the field and charge [44,106,114]. In these tests, a lower density charge [114] and electric field [106,114] were noticed in nanofluids compared to the base fluids. When the stress ceased, the decay rates of the charge were larger in the nanofluids, showing an enhanced capacity to dissipate it [44,105,106]. Again, this appears to confirm the described theory according to the charge and field tendency, as electrons would be able to dissipate by jumping from one particle to another according to the trapping-de-trapping mechanism. The noticed reduction in the mobility of charges in dielectric nanofluids is more evidence that supports this theory [53].

Simulations of the breakdown processes, which have been done using models that try to represent the behavior of nanofluids, have also found differences compared to the base fluids, reaching similar conclusions. Thus, in instant density-of-charge curves, while the base fluids showed a rapid rise in the number of cations and electrons in the propagation wave front, nanofluids also experienced this phenomenon with anions together with a reduction in the presence of free electrons. Moreover, it was seen how the wave front progress was delayed at the same instant it was placed closer to the needle in the nanofluid simulations [188,189], which also happened when plotting the maximal field [10,188]. The electric potential between the electrodes was always bigger in the base fluid simulations [10,188]. These effects were more pronounced when the relaxation time constant of the studied nanofluids was lower [10,188,189]. Here again, the capture capacity of the nanoparticles seems to be a suitable explanation. Nevertheless, some authors cast serious doubts about the universal applicability of the scavenging capacity theory to all the dielectric nanofluids [70].

From these results the current interest of researchers in these kinds of investigations can be justified. However, they have not only focused on the effect of nanoparticles on the dielectric strength, but also on its dependence on other variables, as mentioned in previous sections. In this sense, it has been noticed that variations in the dielectric strength were more accentuated for more concentrated nanofluids, with bigger increases of AC [3,12,19,21,28,36,37,39,41,48,49,57,59,60,64,73,74,80,82,83,86,89,90,98,99,108,116,120,121,122,124,129,131,136], DC [14,28,51,131], and lightning impulse breakdown [14,36,37,67,73,83,85,99,108,120,133,136]. This was also observed in the times to breakdown [83] and PDIV [11,28,73]. Partial discharges, for their part, were reduced as the presence of nanoparticles increased [80,136]. These tendencies are noticeable when comparing research that was carried out with different concentrations ([67,120] and [37,126] and [83]).

Several researchers observed the existence of optimal concentrations at which the dielectric strength started to decrease. These results, collected in Table 18, belong to the lower gaps in the concentrations of the prepared nanofluids in the reviewed papers that are shown in Table 2. The higher availability of traps may enhance electron capture when the concentration of nanoparticles is higher [116], until a determined point at which aggregation results in a reduction of the relative surface and a diminution of the capture capacity [19,36,37,111], especially if stability is affected [21].

**Figure 13:**
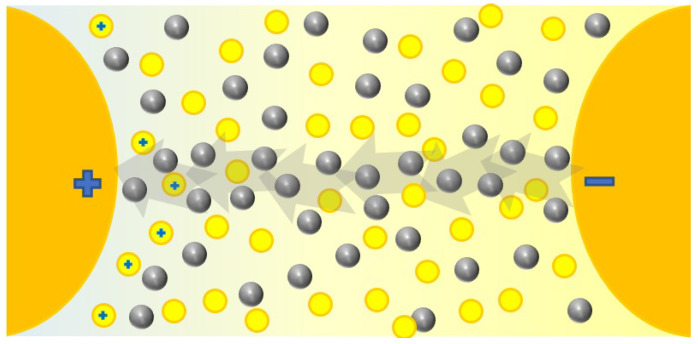
Conductive path of nanoparticles at high concentrations.

In fact, in one of analyzed cases, the optimal concentration corresponded with that where the Z potential was the highest [68]. In other cases, the optimal concentration from point of view of dielectric strength aligned with that of the maximum thermally stimulated current [73,75]. This can even be taken to an extreme when nanoparticle aggregation leads to the formation of “electrically conductive bridges” [13,21,28,38,67,83,102,120,122,129], as the one that is represented in Figure 13, to the point where the breakdown voltage of the nanofluid is lower than that of the base fluid [21]. Another reason could be the fact that these charged aggregates enhance the local electric field, promoting streamer occurrence [28,67,73].

It has already been mentioned that, according to this theory, the size of the dispersed nanoparticles should condition the dielectric strength relative to the surface of the solid fraction, and hence the number of available traps would increase as the size of the particles decreased [52,73,116]. Nonetheless, TSC measurements have shown that the depth of the traps increases as the size of nanoparticles increases [109]. Size may also affect the test results depending on the type of electric stresses that are applied. In the case of slow tests, for AC and DC a larger amount of charge is launched than in the lightning impulse or partial discharges. If the size distributions of nanoparticles have a large enough surface, it could be assumed that there would be traps for every charge. On the contrary, if the particles were too big or in the case of aggregation, the relative surface would be comparatively low, and the positive effect of the particles on the dielectric strength may only be seen for the last two kinds of faults [73,133]. As a result, a dependence on the nanoparticles mean size has been observed [21], with the breakdown voltages increasing with particle size [108,109,133], as well as with smaller sizes when comparing different investigations in which other parameters were alike ([66,108]) ([42,83]) ([37,83]). Thus, a compromise solution between the number and depth of traps together with the stability of the dispersion should be reached by controlling the size distribution in nanofluids, as suggested by the existence of an optimal size for the dielectric properties [133].

The surfactant effect by itself over the base fluids and over nanofluids, has also been addressed. In the first less frequent case, the results seem to depend on the surfactant and base fluid combination, as it has been observed with deterioration [85,88], no effect [88], and improvement [73,85] of the dielectric strength. On the other side, most of the studies reflect an improvement of this parameter in nanofluids due to the presence of surfactants [19,36,41,124]; this can also be seen when comparing the references with similar conditions ([36,42]) ([36,83]). The surfactant that is used conditions the magnitude of the effect [54]. This happens even though the surfactant layers reduce the depth of the traps, as the highest potential is reached at the particle surface due to the reverse polarization of these coatings [109]. This behavior could be explained by the aggregation avoidance that is provided by the surfactants that outweighs its drawbacks and improves the dispersion of the particles [85]. Again, an optimal surfactant concentration seems to exist [19], which fits with the formation of simple or double layers and their consequences for the final behavior of nanoparticles. In this case, it is to be noted that this concentration is approximately 2.7% nanoparticles by mass content. 

Other determinants include the nanoparticle composition and properties, specifically their electric conductivity [10,28,40,86,111,116,121,124,130,131] and hydrophilicity [32,64], base fluid composition [14], and concoction procedure [21,25]. In general, the achieved variation of the dielectric properties depends on the nature of the nanoparticles that are used [128]. Sometimes a mixture of different solid fraction nanofluids show better capacities that the individual nanofluid that is used for the mixture [128].

### 4.2. Dielectric Response Spectroscopy

The concept of dielectric response spectroscopy is comprised of different interdependent parameters that together build a non-destructive alternative for dielectric strength tests using breakdown voltage measurements, such as dielectric relaxation spectrometry [102]. This is based on the polarization of samples with time-dependent electric fields, which causes anisotropy and finally affects the relative permittivity or dielectric constant, volumetric resistivity, and dissipation factor [102], which are controlled. In reality, it is used as a supplement rather than an alternative, since most researchers apply both techniques, although not always completely.

Resistivity (and its opposite, conductivity) had been studied as a function of frequency [40,66,75,95,108]; the resistivity decreases as the frequency increases. The results that were obtained from tests with thermal-dielectric nanofluids were inconclusive, as they present both increases [39,40,48,63,82,104,124,127,130,131] and decreases [53,60,66,80,92,95,111,115,129,130,131] in conductivity, and while the resistivity remained approximately constant at the rated frequencies [37,75,108], at short frequencies they registered higher resistivities than the base fluids [66,75,108]. This could be explained by the number of electrons that were launched at short cycles or high frequencies, as particles might not be capable of capturing them completely [66,108].

The dielectric constant, that represents the capability of a material to become polarized, increases with the presence of nanoparticles [22,40,51,53,59,66,74,75,104,108,112,115,126,129,131] and decreases as the frequency rises [40,51,53,66,75,95,108]. Although not all the references completely support this statement [129], equations for its calculation based on the properties and proportion of the nanofluids’ components confirm this tendency [123,124,126], and indicate that it is larger with conductive particles [22,51,126]. This also seems to depend on the size, as some theories relate to smaller particles with higher permittivity [108].

Finally, the dissipation factor (tan δ) analyzes the dielectric losses of an insulator by representing how much the fluid’s impedance deviates from the reactive component due to conductance and polarization losses [54,66]. It is a measurement of insulant quality, as it gets worse, i.e., its value increases, when pollutants, moisture, or products of degradation are present. In these nanofluids it shows erratic behavior, as with resistivity, both increasing [39,40,48,59,60,95,97,108,112,113,115,126,127] and decreasing [51,53,66,75,80,95,113,129], compared to the base fluids, and decreasing as the frequency increases [40,51,53,59,75,95,108,112].

According to the previous explanation, as they are interlinked, tan δ might increase when the conductivity or dielectric constant increases, as this would suggest conductivity or polarization losses, respectively. Despite this, dielectric oils are low polarity fluids, so the contribution of these losses is negligible, and as such, the prevailing effect is from resistivity changes [40,66,75,80,111,130,131]. Variations in the resistivity and dissipation factor of different signs appeared together in several of the papers that we examined [39,40,48,53,95,111,124,129,130,131].

These three parameters are affected by the concentration of particles [95], which has been shown to accentuate their tendencies [16,22,39,48,51,53,59,60,63,80,97,112,114,115,124,126,127,129,131]. The presence of surfactants also conditions the magnitude of these parameters [53]. Permittivity depends on the size of the solid fraction as they have been shown to vary contrarily [108]. Considering the particles’ composition, it seems logical that those which are more conductive translate into less resistive nanofluids, but there are results which both support [95,130,131] and deny [66,82,104] this behavior.

## 5. Nanofluids Application

Once the positive trends in the properties of thermal-dielectric nanofluids are checked, the focus of research will move to demonstrate their applicability in actual equipment. This will depend on their stability, environmental influence on fluid behavior and properties, relationship with other components, and natural evolution with time under working conditions.

For this, it must be taken into account that nanofluids will have to support high temperatures, the presence of moisture and magnetic fields, as well as possible faults and stresses, all under the flow conditions that are determined by the type of cooling circuit.

### 5.1. Environmental Conditions

It is compulsory to know how environmental conditions in equipment would affect the nanofluid under use, especially due to the presence of nanoparticles with respect to the base fluid. In this sense, some researchers have committed part of their efforts to this objective.

It is known that moisture has perverse effects for dielectric properties since, as a conductive and polarizable substance that promotes ionization and electron appearance, its presence reduces the dielectric strength of the fluid, as seen in AC breakdown voltage [1,7,65,68,98,106,116,121,134] and PDIV [65,134] tests, although it does not affect the lightning impulse results. With respect to the influence of nanoparticles, it has been noticed that when the dispersion is prepared with hydrophilic particles, they tend to withdraw water molecules from the base fluid, and as a result an enhancement of the dielectric strength is noticed [1,28,98,106], even more with higher concentrations [78,98] or moisture content [1,64,98,106], to a point [7]. Additional evidence for this came from treatments with hydrophobic surfactants, as dielectric strength and gravimetric weight loss were less, which is what was read as lower quantities of water that was captured [64]. Also, from TSC tests, as wetter dielectric nanofluids were able to release higher currents, so their capacity to capture electrons might be higher [106]. Other similar behaviors were found in [134] with ferrofluids over breakdown voltage and PDIV.

Temperature is a key factor. As cooling fluids get hot, especially in some points of transformer geometry, their dielectric strength increases due to their lower viscosity and higher energy of electrons, which makes streamer formation difficult [131]. In nanofluids this tendency is the same [56,78], also promoted by the enhancement of the Brownian movement of particles, hindering the aggregation and formation of preferential ways. This higher movement also hinders their polarization and promotes electrical conductivity, which is seen in lower permittivity [59,129,131] and resistivity [40,53,129,131] and higher conductivity [53,63,82] and dissipation factor [40,53,59,129,130,131]. Although the behavior of the nanofluids were better at working temperatures, these also negatively affect the stability of suspension of particles, promoting sedimentation, that may be offset by forced flow regimes.

Another environmental factor to consider is the presence of magnetic fields in the equipment, especially when magnetic nanofluids are under study. Their magnetic properties are also affected by the presence of nanoparticles, and these changes translate finally into stability and thermal-dielectric properties. To measure it, magnetic susceptibility is useful, as it is the ratio between magnetization M and the magnetic field H, represented in magnetization curves M/H, that are obtained from magnetometers at different magnetic fields and temperatures.

Comparing these curves, it can be seen that the susceptibility of magnetic nanofluids grows with respect to the base fluids, even more with bigger concentrations, and that this parameter decreases as temperature rises [20,58,97,180,191]. When surfactants are applied, this susceptibility decreases, due to a higher presence of non-magnetic material in solid fraction [21], which happens in other revised references [23,107,149,152,169]. It has also been seen that this presence affects the magnetic behavior as it reduces interactions between the particles, and as a result at rated temperatures the nanofluid behaves as a superparamagnetic material, without hysteresis phenomenon, so the remnant field is zero when the external field disappears [20,21,69]. As the particles are kept separated from each other, their magnetic domains recover their aleatory distribution, what did not happen at low temperatures or without surfactants in the proper amount. This capacity would be useful to check what is the optimal concentration of surfactant that is capable of maintaining separated particles [20,139].

When the external field is not zero, the particles tend to align with field lines, according to their dipoles, which translates in more powerful interactions, aggregation, or the formation of more orderly structures [8,37,102], unless their aleatory Brownian movement hinders these phenomena. This has been supported by acoustic spectroscopy tests, where acoustic attenuation is measured as it varies with aggregation, showing their results anisotropy in attenuation with respect to the field direction and higher values as bigger in the magnitude of the field, and how it decreases as the temperature grows [33,167]. Moreover, these tendencies continued when the maximal field was removed, as a proof of hysteresis occurrence due to aggregation. Nevertheless, experimental tests have not found variations of concentration when the samples are submitted to magnetic fields due to aggregation [9,13].

This behavior when exposed to magnetism must have influence on other properties of magnetic nanofluid. First, to their cooling capacity. As in transformers, the dielectric fluid is under nonuniform magnetic fluid, and similarly when speaking about temperature distribution, they would appear as magnetic forces when the particles were magnetized [102,180,182]. This was confirmed by simulations [34,122,125,138], depending on the field magnitude and temperature, that in the radial direction [180] would contribute to convective cycles from coldest zones to coils [16,67,69,97,120,126,138,180,182], and whenever in their proximity the particles were demagnetized by reaching temperatures over Curie’s, at which time the particles lost their magnetic susceptibility [145,180,182]. Since its contribution is considerable, it is recommended high pyromagnetic coefficient (variation of magnetization with temperature [180,182]), low Curie’s temperature, large thermal conductivity, a maximal saturation magnetization, and low viscosity are all combined with enough concentration of particles [16,180,192]. The appearance of Lorentz forces as charged particles move under a variable magnetic field can also contribute to convection [16].

For its part, thermal conductivity could be enhanced by the alignment of particles, creating again preferential ways when concentrations and magnetic field magnitude are suitable; an anisotropy that supposes an increase of conductivity when thermal gradient and field are parallel and a decrease when perpendicular if a conductive bridge is completed. If not, this enhancement is in every direction, but even more on a field one [8].

Finally, the dielectric properties seem to be improved by external magnetic fields [13,16,17,133], depending on the field direction [13,133], except in the case of formation of the conductive bridge [38]. In the case of permittivity, this occurs on the contrary, as it rises when electric and magnetic fields are parallel and sinks when they are perpendicular [123].

### 5.2. Neighbouring Relationship

It is important to consider the relationship between dielectric oils and other transformer components. The dielectric papers and pressboards that protect the coils must be impregnated by the oils while maintaining or improving their insulating capacities [80], [111], but these are not the only requirements. The conservation of cellulosic components must be ensured; thus, the surrounding medium must not be aggressive from a chemical point of view. Another reason why the relationship between the fluid and paper is relevant, is that progressive surface discharges at their interface are considered to be the main cause of failure in transformers. This kind of fault causes cellulosic deterioration and the appearance of carbonized traces, which facilitates dielectric breakdown if the stress is high enough [43].

For this reason, some researchers have studied samples of nanofluids and base fluids that are in contact with previously dried dielectric papers [24,43,44,55,96]. Firstly, they realized that particles tend to migrate toward the porous paper, as reflected in microscopy images [44]. A consequence and evidence of this was the change in the magnetism of the paper, from paramagnetism when dry, to superparamagnetism due to the presence of magnetic particles [9]. Once washed with oil, the paper returned to its initial condition, so the particles were not bonded to the paper. Other authors, nevertheless, have noticed the existence of bonds between surfacted nanoparticles and paper structure using the surfactants as bridges [55].

The particles also had an effect on the moisture. It is known that the presence of moisture is higher in cellulosics than in oil, but the water content of paper that is impregnated with nanofluid is comparatively low. This may have positive consequences on the dielectric properties of paper, as the increase in moisture translates to a larger dissipation factor at rated frequencies [193]. Their dielectric strength, measured with equipment that is designed for paper samples using techniques that are similar to those that have already been mentioned, is also favored, according to results that were obtained for AC, DC, and lightning impulse breakdown voltages (+10 to 67%, +6 to 9%, and +19 to 31%, respectively), PDIV (+6 to 12%), and progressive discharge voltage (flashover) at the interface (+12 to 15%) [43,44,55,96]. The streamers are smaller, slower, and easier to dissipate with the presence of nanoparticles, even more as their mean diameter is shorter [24]. The theory of traps is again capable of explaining these results due to the presence of particles on the surface of the paper. The PEA and TSC tests also support this as the electric field is more homogeneous and lower; this also happened with the charge density, which was dispersed faster [43,44]. The TSC tecniques also revealed a deepening of the potential traps of the paper due to the presence of nanoparticles, which were more noticeable as the diameters were larger [55]. The dissipation factor also improved as predicted [21,65], especially as the concentration of particles increased [21], but not in all the references [55]. The relative permittivity seems to be enhanced, especially with larger nanoparticles [55].

### 5.3. Aging Assessment

These tests have not only checked the initial stage of the nanofluid-paper combination, but also how these systems evolve with time under working conditions where they are submitted to electrical, chemical, mechanical, and thermal stresses. These stresses lead to the progressive degradation of the hydrocarbon chains in these components, and the liberation of by-products such as water, acids, sludges, gases, and free electrons, which in turn feedback the degradation. These substances harm the capacities of dielectrics, since they act as a focus of charge accumulation and distort the electric field, to a point that they do not fulfil the requirements, shortening the life expectancy of dielectrics, and necessitating their replacement to avoid isolation failures that endanger the installations and their functioning.

Nevertheless, degradation processes take place over several years, so faster techniques to estimate the life expectancy of nanofluids and impregnated cellulosics became necessary. For this purpose, samples were submitted to accelerated thermal aging in different mass ratios of fluid/paper (10:1 or 20:1), dried and degassed, with presence of copper, and in an inert atmosphere, at temperatures several grades above the mean operating temperature in transformers [193,194]. In these conditions, with same elements found in actual transformers, the samples needed less time to suffer the complete process of degradation. Periodically, parameters that were representative of the degradation progress such as moisture, dissolved gasses, acidity, and degree of polymerization, were measured. Subsequently, by means of equations [16,46,68,195], the equivalent time of every measurement can be estimated at a determined temperature.

The results of tests that were done with aged samples of nanofluids and cellulosics, for periods between 1 and 500 days, at temperatures from 90 to 185 °C, reflect similar behavior to those that were seen in fresh nanofluids. The dielectric strength of an aged base fluid is enhanced by the presence of nanoparticles (Table 19) and the increases that were seen could be larger than those with fresh oils in AC [46,100], lightning impulse breakdown voltage [40,45], and PDIV [45,46,103] tests. This situation again supports the theory of traps, as this effect would be explained by the lower degradation of nanofluids resulting from the capture of free electrons [45], with the additional contribution of water withdrawal when that occurred [68,69]. In other cases, the increase of moisture during aging could explain the diminution of the dielectric strength [86]. The degree of polymerization (DP) of aged paper samples, which represents how well their molecular structure has been conserved, has also shown improvements [132] and deterioration [86,196], that were explained respectively by the supposed capacity of the nanofluid to delay the degradation, and by the particles performing as hot-spots on the surface of the paper. The analysis of the degradation gases produced that were during the ageing process of nanofluids was also not conclusive [50]. The acidity and other of the indicators of degradation, tends to rise in the presence of nanoparticles [86].

In this same way, the dielectric strength of transformer oils is harmed by successive electrical faults. This is why another kind of degradation test consists of submitting the samples to consecutive breakdowns and checking their dielectric strength. In the cases of lightning impulse [120] or AC [21] voltages, it has been noticed that the samples are capable of maintaining their dielectric strength, with increases over the base fluid in the same conditions until 70%, and around 30% with respect to the fresh base fluid [120]. The reason that was given for this was that as discharges occurred through particles, not the fluid, it was kept protected from the discharges effects [21].

## 6. Conclusions

Over the last two decades, since the rise of the idea of their application in power transformers, researchers have dedicated a great amount of effort to the search for efficient thermal-dielectric nanofluids. The main objective has always been to improve the performance of actual equipment and to allow the design of smaller, but more powerful transformers in the future. To achieve this, it was first mandatory to demonstrate the beneficial effect of nanoparticles on their thermal and dielectric properties. Secondly, the parameters that were responsible for this effect had to be identified and optimized. Finally, the nanofluids needed to fulfil the applicability requirements had to be identified.

This review collates much of the information that has been published by researchers on this subject, allowing us to reach conclusions about these issues, such as the confirmation of the higher thermal and dielectric capacities of nanofluids that were prepared with dielectric cooling fluids. Significant relative increases, frequently between 20 and 50%, have been noticed in the thermal conductivity and breakdown voltages of nanofluids compared to their corresponding base fluids. However, variations in the first property pale in comparison to the second, taking into account the magnitude of the base fluid’s thermal conductivity. Nevertheless, increases in the conductivity were sufficient to improve the global heat transference or convection coefficients, according to the scarce experimental data that are available. The consecution of improvements in both the thermal and dielectric properties is required in order to justify the addition of nanoparticles to transformer coolant fluids.

The enhancement of these properties can also be optimized with proper design and preparation of the nanofluids; this dependence has been verified for parameters such as the concentration, size, shape, and composition of the nanoparticles. The first characteristic is of particular importance, as both the thermal and dielectric capacities have been shown to grow with the concentration of the nanoparticles. This is why most researchers have studied its effects on the final properties of the nanofluid, noticing the existence of optimal concentrations above which an excessive presence of the solid fraction is detrimental. Nevertheless, this limitation on nanofluids is not adverse, as it also comes from stability constraints that are related to the applicability of nanofluids. Thus, fortunately, the requirements for stability are not in competition with those for the thermal-dielectric properties, as the range of concentrations is limited by both.

Stability is the first condition that is required for dispersions, so their improved properties persist over time; it requires their control, as well as treatments that make the particles suitable for dielectric base fluids. Again, the use of surfactants does not appear to be detrimental to other aspects of their performance. The results show that most stabilities are under the mean residence time in actual transformers, although they have been studied under static conditions, not in working cooling circuits. A bigger effort in this sense is needed.

The great number of existing variables in the preparation processes of thermal-dielectric nanofluids makes it challenging to find an optimized methodology which is suitable for the production of dispersions for transformer applications. This includes not only the production of nanoparticles, their characteristics, properties, and surface treatments that are mentioned above, but also how are they carried out, such as the base fluid, as well as how the particles are added and dispersed into it.

Several processes have already been established. According to vast majority of these, the nanofluids were prepared following a two-step method, probably due to the difficulty of using mineral oils or natural esters as the synthesis medium for nanoparticles; or the dispersion of small and spherical nanoparticles in low concentrations, by a combination of mechanical shaking and ultrasound.

Considering the methods that are still under discussion, from the information that was collected here, the beneficial effects of the use of surfactants can be observed when they are used in proper concentrations and chosen specifically for a determined nanofluid based on its components. The length of organic chains, stability against temperature, and the design of the surfactantion procedure all affect the results. The contact between the particles and surfactants must be ensured, for example by putting them together before the addition of the base fluid. Similarly, enhanced bonds between the nanoparticles and surfactants can be achieved by applying heat during the treatment. To avoid the formation of double surfactant layers, excess surfactant should be removed by washing with organic solvents and recovering the particles, a step which can be useful to select those particles to add to the base fluid. Once a surfacted powder is obtained, and considering the final use of the nanoparticles, drying under a vacuum or inert atmosphere is convenient.

The dispersion of particles must be optimized, beyond the combination of different mechanisms. Heating of the samples during large sonications may result in the breaking of bonds between the surfactants and solid surfaces; this can be prevented by periodic stops or cooling systems. Another drying step, to remove moisture that is absorbed during handling, is also useful for the elimination of bubbles.

**Figure 14:**
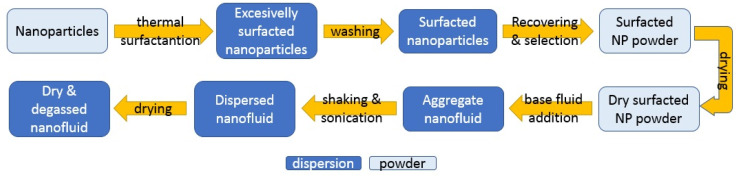
Preparation method for dielectric nanofluids with improved stability.

Thus, a suitable process for the preparation of nanofluids for the cooling and isolation of transformers, from the point of view of stability, is shown in Figure 14.

Each step of this proposed general procedure has been independently shown to be effective, but they must be tested together while considering the recommendations with respect to the nanoparticle, surfactant, and nanofluid characteristics. Different compositions, concentrations, base fluids, surfactants, dispersion times, and temperatures should also be tested, adjusting the method for every combination. Of course, once the effectiveness of this methodology for the stability of nanofluids has been demonstrated, these stable nanofluids must be submitted to characterization tests to ensure that their enhanced properties remain. Their existence and perdurability, intimately linked to durable suspensions, are requisites to support the applicability of nanofluids in transformers. Time-evolution studies of the properties may also be needed.

Considering applicability, despite the current stability of dispersions, other factors have shown positive tendencies. It has been noticed that environmental conditions during work in the actual equipment do not represent problems, but advantages for these nanofluids, as their properties would be even enhanced with respect to the base fluids with the same conditions of temperature or moisture, except in the case of higher temperatures (over 80 °C) that harm stability or surfactants integrity or magnetic fields that promote aggregation. Similarly, it occurs with relationship between nanofluids and cellulosics, as the presence of nanoparticles also seems to improve cellulosics properties, and the resistance to aging that is caused by stress, at least in the case of oils.

These extra characteristics of dielectric nanofluids, if confirmed using samples with demonstrated thermal-dielectric properties and stability, would represent a definitive step in this field. So, during following years, research should focus on the consecution of this efficient preparation methodology for nanofluids, and the confirmation of their theoretical properties. Once this is achieved, it will be necessary to check the behavior of the thermal-dielectric nanofluids in pilot plants and transformers under working conditions.

In parallel, other aspects which have been comparatively less demonstrated than others, and parameters whose effects are still uncertain, such as size or shape, should remain under investigation in an attempt to confirm their influence, especially over global heat transfer capacities. The range of particle compositions may be broadened. For this, the synthesis of ad hoc particles may be helpful. In the same sense, efforts to demonstrate whether the presence of particles definitively promotes or decelerates paper aging must be strengthened.

## Figures and Tables

**Table 1:** Mean and maximum distribution size of nanoparticles that are used in dielectric nanofluids.

Size	Reference
Mean size	
<10 nm	[7,8,9,10,11,12,13,14,15,16,17,18,19,20,21,22,23,24,25,26,27,28,29,30,31]
10–20 nm	[3,18,23,24,25,27,28,31,32,33,34,35,36,37,38,39,40,41,42,43,44,45,46,47,48,49,50,51,52,53,54,55,56,57,58,59,60,61,62,63,64]
20–30 nm	[30,55,65,66,67,68,69,70,71,72]
30–40 nm	[27,73,74,75,76,77,78,79]
40–50 nm	[28,55,61,74,78,79,80,81,82,83,84,85,86]
50–100 nm	[32,52,75,86,87,88,89,90,91,92,93]
>100 nm	[74,91,92,94,95,96]
Distribution larger size	
<10 nm	[26,97]
10–20 nm	[26,37,42,46,47,98,99,100,101,102,103,104,105,106]
20–50 nm	[3,27,34,83,107,108,109,110,111,112]
50–100 nm	[89,90,111,112]
>100 nm	[12,20,75,91,113]

**Table 2:** Nanoparticle content in dielectric nanofluids in the revised literature.

Concentration	Reference
% Volume	
<0.05%	[20,26,32,49,78,85,95,98,109,111,114,115]
0.05–0.1%	[24,54,56,65,85,94,104,115,116,117,118]
0.1–0.5%	[15,18,56,73,74,82,92,118,119]
0.5–1%	[17,25,27,63,67,77,81,88,92,101,103,104,105,118,119,120,121,122]
1–5%	[8,13,33,34,38,61,76,94,97,123,124,125]
5–10%	[87,126]
20–40%	[83]
% Weight	
<0.01%	[7,19,32,66,79,84,93,95]
0.01–0.05%	[21,31,49,57,86,89,91,108,111,127,128,129]
0.05–0.1%	[12,30,57,64,70,80,98,116,128,129,130,131]
0.1–0.5%	[51,56,57,58,71,128]
0.5–1%	[51,58,66,71,132]
1–5%	[29,56,58,71]
>5%	[58]
g/L	
<0.1 g/L	[3,35,36,37,52,59,68,95,113]
0.1–1 g/L	[11,14,18,28,39,48,49,50,52,53,59,60,62,65,72,90,96,110,112,133,134]
1–10 g/L	[42,60,83,135,136]
10–50 g/L	[41,43,44,74]

**Table 3:** Composition of nanoparticles that were used in references.

Nanoparticle Composition	Reference
Magnetite (Fe_3_O_4_)	[1,7,8,9,10,11,13,14,16,17,18,19,28,34,38,39,40,48,52,55,56,58,59,62,66,67,68,69,75,83,97,102,108,109,120,123,125,126,131,132,133,134,136,137,138,139]
Other Fe oxides	[20,21,33,40,49,88,112,113,122]
Polymetallic ferrites	[23,124]
Titania (TiO_2_)	[3,24,25,30,36,37,41,43,44,45,46,47,51,54,60,65,68,72,74,78,83,86,89,91,92,101,103,104,105,106,111,112,113,114,121,128]
SiO_2_	[27,28,31,32,40,42,50,52,53,64,88,98,121,140]
Al_2_O_3_	[10,40,42,45,50,51,52,61,71,72,77,79,81,83,85,88,91,93,94,96,110,112,113,118,119,128,135]
ZnO	[40,63,70,74,78,81,86,95,121]
CuO	[76,94,112]
ZrO_2_ or CeO	[29,57,95,111]
Bimetallic oxides	[78,92]
Cu, Al or Ag	[27,87,94]
AlN, BN or SiC	[61,73,79,80,82,88,117,131]
Carbon Nanotubes (CNT)	[26,27,116]
Fullerene	[12,27,30,31]
Graphite/Graphene/Diamond	[26,127,129,130]

**Table 4:** Thermal conductivities of nanoparticles and dielectric oil.

**Bulk material**	SiO_2_	CuO	Fe_3_O_4_	Fe_2_O_3_	Al_2_O_3_	TiO_2_	ZnO
**k (W/m·K)**	10.4	76.5	1.39	80	36	8.4	13
**Bulk material**	AlN	SiC	Diamond	CNT	Fullerene	Mineral Oil	
**k (W/m·K)**	140	35	2200	3000	0.4	0.1	

**Table 5:** Classification of nanoparticles in function of their electric conductivity.

**Conductive**	Fe_3_O_4_
	Fe_2_O_3_
	ZnO
	Graphene
**Semiconductive**	TiO_2_
	CuO
	ZrO_2_
	SiC
**Insulating**	SiO_2_
	Diamond
	Al_2_O_3_
	BN
	AlN

**Table 6:** Temperature during nanoparticle synthesis reaction in studied research.

Reaction Temperature (°C)	Reference	Nanoparticles Produced
Room Temperature	[8,149,150,152,153,157,158,159]	Fe_3_O_4_-Fe_2_O_3_
<100	[20,34,58,108,109,116,123,124,137,145,154,155,164,165]	Fe_3_O_4_-Fe_2_O_3_-Cu-Bi_2_Te_3_-Graphene
100–200	[23,25,101,133,146,151,161]	TiO_2_-Fe_3_O_4_-Fe_2_O_3_
200–300	[57,139]	CeO_2_-Fe_3_O_4_
300–500	[3,7,19,21,55,108,109]	Fe_3_O_4_-TiO_2_-ZnO
500–1000	[65,96,142,162]	TiO_2_-Al_2_O_3_-CNT

**Table 7:** Surfactants that were used in thermal-dielectric nanofluids.

Surfactant	Reference	Nanoparticle Treated	Base Fluid
None	[1,12,36,42,49,64,82,86,99,100,119,130]	Fe_3_O_4_-Fe_2_O_3_-SiO_2_-Fullerene-Al_2_O_3_-TiO_2_-AlN-Graphene	Mineral oil Natural Ester
CTAB	[19,53,85,89,90]	Fe_3_O_4_-TiO_2_	Mineral oil
SDBS	[110]	Al_2_O_3_	Mineral oil
SDS	[85,129]	Al_2_O_3-_Graphene	Natural ester
Span 80	[10,30,88]	Fe_3_O_4_-Fe_2_O_3_-SiO_2_-Al_2_O_3_ -SiC-TiO_2_	Mineral oil
Oleic acid	[7,8,10,13,14,16,18,20,21,24,25,28,33,34,38,51,54,55,56,58,61,63,65,66,67,69,73,75,84,85,87,91,93,96,101,102,108,109,113,116,123,124,133,136,138]	Fe_3_O_4_-Fe_2_O_3_-SiO_2_-Al_2_O_3_-Cu-TiO_2_-AlN-CNT	Mineral oil Natural ester Synthetic ester
Acetic acid	[54]	TiO_2_	Mineral oil
Hexanoic acid	[54]	TiO_2_	Mineral oil
Lauric acid	[87]	Cu	Mineral oil
Stearic acid	[10,36,41,43,44]	Fe_3_O_4_-Al_2_O_3_-TiO_2_	Mineral oil
Silane	[36,41,44,64,70,73]	SiO_2_-TiO_2_-ZnO-AlN	Mineral oil Natural ester

**Table 8:** Surfactant concentrations that were found in the references.

Surfactant Maximum Concentration						
g/L	0.00075			2.5		
	[53]			[66]		
%vol	<0.025%	0.06%	0.25%	1.5%	4%	10%
	[85]	[93]	[51,88]	[90]	[34]	[91,113]
mL/g	0.026	0.05		2		10
	[21]	[51]		[11]		[20]
%w	<1%	<1.5%	2–3%		22%	40%
	[110]	[89]	[19]		[87]	[30]

**Table 9:** Classification of references as a function of the base fluid that was used.

Base Fluid	Reference
Water	[27,87,107,142,153,162,163,168,170,171,173,174,175,176,177,178,179]
Mineral oil	[1,3,8,9,10,11,12,13,14,15,16,17,18,19,24,25,26,28,29,30,31,32,33,34,35,36,37,38,39,40,41,42,43,44,45,46,47,50,54,57,61,62,63,64,65,67,68,69,70,71,72,73,74,76,80,81,82,83,87,88,89,90,91,94,96,97,98,99,100,101,102,103,104,105,106,110,111,112,113,116,117,118,119,120,121,122,123,124,128,130,131,133,134,135,136,137,138,140,143,178,180,181,182]
Synthetic ester	[14,53,59,113]
Natural ester	[7,20,21,48,50,51,52,55,60,66,75,78,79,84,85,86,92,93,95,108,109,114,115,117,129,132]
Paraffinic oil	[33,88]
Wasted oil	[22,127]
Ethylene Glycol	[33,168,177,178,183]
Hydrocarbons	[33,107,125]
Organic solvents	[74,107,165,178]
Gas to Liquid (GTL)	[58]

**Table 10:** Homogenization times during nanofluids preparation.

Time	Sonication	Stirrer Shaking	Nanoparticle	Base Fluid
<30 min	[21,24,28,66,67,73,74,92,120,132]	[21,29,32,49,51,60,64,67,68,72,89,91,98,110,113]	Fe_2_O_3_-Fe_3_O_4_-TiO_2_-SiO_2_-Al_2_O_3-_AlN-BaTiO_3_	Mineral oil–Natural ester–Synthetic ester
30 min−1 h	[55,70,80,95,112,127]	[30,31,52,53,56,78,79,80,85,92,93,95]	BN-CuO-Fe_3_O_4_-Fe_2_O_3_-Fullerene-ZnO-ZrO-SiC-SiO_2_-TiO_2-_BaTiO_3-_Al_2_O_3_-Graphene	Mineral oil–Natural ester–Synthetic ester–Waste oil
1–2 h	[27,30,31,32,48,64,68,78,79,91,93,98,110,128]	[57]	ZnO-SiO_2_-TiO_2_-Al_2_O_3-_Fe_3_O_4_-CeO_2_-SiC-BaTiO_3_-Carbon nanotubes-fullerene	Mineral oil–Natural ester–Paraffinic oil
2–6 h	[25,52,53,72,85,86,89,113,130]	[25,129]	TiO_2_-BN-Al_2_O_3_-SiO_2_-ZnO-Graphene	Mineral oil–Natural ester–Synthetic ester
6–12 h	[49,57,87]	[128]	Cu-Fe_2_O_3_-TiO_2_-Al_2_O_3_-CeO_2_	Mineral oil–Natural ester
12–24 h	[60]	[34]	TiO_2_-Fe_3_O_4_	Mineral oil–Natural ester

**Table 11:** Nanofluid stability classification regarding Z potential.

Z Potential (mV)	Stability
0–15	Little or no stability
15–30	Some stability but settling lightly
30–45	Moderate stability
45–60	Good stability, possible settling
>60	Very good stability, little settling likely

**Table 12:** Stability lasting of nanofluids in the analyzed research.

Stability	References	Base Oil	Nanoparticles
Less than 1 day	[84,108]	Mineral oil–Paraffinic oil	Al_2_O_3_-Iron oxides
1 day or more	[9,11,14,18,27,84,88,153]	Mineral oil	Fe_3_O_4_-CNT-SiO_2-_SiC-Al_2_O_3_
1 month or more	[7,32,48,65,80,112,125,130,141,181]	Mineral oil–Paraffinic oil–Natural ester	SiO_2_-Fullerene-CNT-Ag-Fe_3_O_4_-AlN-BN-Graphene
6 months or more	[23,68,105],	Mineral oil–Natural ester	AlN-Fe_3_O_4_
1 year or more	[12,17,28,34,36,37,47,104],	Mineral oil–Natural ester	Fe_3_O_4-_Fe_2_O_3_-Fullerene-CeO-TiO_2_
2 years or more	[19,30]	Mineral oil–Natural ester	Fe_2_O_3_-TiO_2_

**Table 13:** Maximal thermal conductivity variation in oil-based cooling nanofluids with respect to the base fluid.

Maximal Δk (%)	Reference
<0	[60,64]
0–5	[25,30,49,59,88,91,113,131]
5–10	[27,73,88,91,113]
10–20	[115,129]
20–50	[8,21,26,61,87,143,178,181]
50–100	[124,130]

**Table 14:** Nanofluids characteristics and their effects on thermal conductivity of base fluids.

Ref.	Base Fluid	Nanoparticle	Concentration	Size	k Variation
[8]	Engine oil	Fe_3_O_4_	<5%v	---	≈40%
[20]	Mineral oil	TiO_2_	<0.1%w	25 nm	≈0%
		Fullerene		70 nm	
[21]	Vegetal oil	Fe_2_O_3_	<0.014%w	10 nm	45.00%
[22]	Waste oil	SiC	<0.3%v	30 nm	23.00%
		TiO_2_		10 nm	
[25]	Mineral oil	TiO_2_	0.075%v	18 nm	1.20%
[26]	Mineral oil	NTC	<0.05%v	15 nm	25.00%
		Diamond		6 nm	20.00%
[27]	Paraffinic oil	CNT	0.5%v	10–30 nm	8.50%
[59]	Synthetic ester	Fe_3_O_4_	0.05 g/L	10 nm	2.7%
[61]	Mineral oil	Al_2_O_3_	<4%v	13 nm & rods	20.00%
		AlN		50 nm	20.00%
[64]	Mineral oil	SiO_2_	<0.1%w	15 nm	−1.60%
[73]	Mineral oil	AlN	<0.16%v	40 nm	7.00%
[87]	Mineral oil	Cu	<7.5%v	100 nm	43.00%
[88]	Mineral oil	Al_2_O_3_	<1%v	<80 nm	≈7%
		SiO_2_		<100 nm	≈3%
		SiC		<80 nm	≈10%
	Synthetic oil	Al_2_O_3_		<80 nm	≈5%
		SiO_2_		<100 nm	≈3%
		SiC		<80 nm	≈5.5%
		Fe_2_O_3_		<100 nm	≈3%
[91]	Mineral Oil	Al_2_O_3_	0.02%w	<130 nm	≈7%
		TiO_2_	0.02%w	< 110 nm	≈2%
[113]	Mineral Oil	Al_2_O_3_	0.02 g/L	< 110 nm	8.27%
		TiO_2_	0.02 g/L	< 110 nm	4.08%
[115]	Natural ester	BN	<0.1%v	---	11%
[124]	Mineral oil	Mg_0.40_Mn_0.60_–xNi_x_Fe_2_O_4_	<4%v	---	58.00%
[129]	Natural ester	Graphene	<0.05%w	---	36.4%
[130]	Mineral oil	BN	<0.1%w	---	76.00%
[131]	Mineral oil	BN	<0.1% w	50 nm	≈1%
		Fe_3_O_4_		20 nm	≈0.5%
[143]	Mineral oil	Ag	<0.72%w	20 nm	≈30%
[177]	Mineral oil	Al_2_O_3_	5%v	---	38.00%
[181]	Mineral oil	CNT	<0.5%w	10–20 nm	22.70%

**Table 15:** Maximal variations of AC breakdown voltage of nanofluids with respect to the base oils.

Maximal Variation (%)	AC BDV	AC BDV Low Probability
<0	[26,42,56,73,94,190]	[56,190]
0–5	[36,79,80]	[29]
5–10	[19,74,79,84,140,159]	[79,100]
10–20	[29,35,36,37,45,59,74,78,79,92,100,103,104,105,128,136]	[7,79]
20–50	[1,3,12,21,25,35,36,39,48,49,60,62,64,66,69,74,75,78,81,86,89,92,93,94,98,99,103,108,109,111,114,116,120,121,128,129,134]	[28,45,93,103,104]
50–100	[57,64,95,124]	[12,21,89,98,99,190]
>100	[17,90,122]	-

**Table 16:** Maximal variations of the breakdown voltage and time to breakdown of nanofluids with respect to the base oils in lightning impulse tests.

Maximal Variation (%)	BDV + Impulse	Time to Streamer	BDV–Impulse (-)	Time to Streamer (-)
<0	[40]	[40,79]	[1,14,40,66,73,85,108,132]	[40,66,79]
0–10	[29,35,79,83,135]	[83,85]	[79,126,135]	[40,79,85,126]
10–25	[14,36,37,40,42,45,75,83,85,104,105,111,120,132]	[37,40,66,83,85,135]	[40,83,133]	[83,85,135]
25–50	[10,46,54,66,73,79,99,100,132,133,136],	[126]	[40,79,136]	[1,83,126]
50–100	[1,108,126]	[40,47,54,104,133,136]		[40]
>100		[1,10,126]		

**Table 17:** Maximal variations of the partial discharge inception voltage and magnitude of the discharges in nanofluids.

Variation (%)	PDIV	PD (-)
0–5	-	[32]
5–10	[45,46,62,103,104,114]	-
10–20	[31,32,72]	[32,45,93]
20–50	[1,31,73,79,93]	[31,80,104]
50–100	[28,79]	[31,72,114]

**Table 18:** Optimal concentrations of nanofluids with respect to their dielectric properties.

Optimal Concentration	Reference
% Volume	
<0.05%	[49,74]
0.05–0.5%	[67,73,120]
0.5–1%	[38,122]
1–5%	[8,13,33,34,38,61,94,97,123,124,125]
5–10%	[87,126]
20–40%	[83]
% Weight	
<0.01%	[3,19,21,36,83,89,111,116]
0.01–0.05%	[11,28,49,83,86,116,129,134,136]
0.05–0.1%	[57,116]
>0.1%	[51]
g/L	
<0.1 g/L	[3,11,19,36,83,89,95,111]
0.1–1 g/L	[21,28,39,49,59,60,83,116,133,134,136]
1–5 g/L	[74]

**Table 19:** Properties’ maximal variations showed by nanofluids vs. aged base oils in the analyzed works.

Ref.	AC BDV	AC BDV at Low Probability	Lightning Impulse BDV	PDIV
[3]	14%			
[9]	33.3%	-	-	-
[45,47,103]	7.7%	11.4% at 1%	47.1%	12.2%
[46]	40%	-	30%	10%
[68]	200%	-	-	-
[73]	17.5%	-	49.4%	27.9%
[81]	39.3%			
[86]	28%			
[100]	10.7%	19.5% at 5%	21.5%	

## References

[1] Segal V., Hjortsberg A., Rabinovich A., Nattrass D., Raj K. AC (60 Hz) and impulse breakdown strength of a colloidal fluid based on transformer oil and magnetite nanoparticles. Proceedings of the IEEE International Symposium on Electrical Insulation (ISEI).

[2] Choi S.U.S.S., Eastman J.A., Choi S.U.S.S. (1995). Enhancing thermal conductivity of fluids with nanoparticles. Proceedings of the ASME International Mechanical Engineering Congress & Exposition.

[3] Fallah-Shojaie A., Tavakoli A., Ghomashpasand M., Hoseinzadeh S. Experimental evaluation on the dielectric breakdown voltage of fresh and used transformer oil mixed with titanium dioxide nanoparticles in the Gilan electrical distribution company. Proceedings of the Iranian Conference on Electrical Engineering (ICEE).

[4] Gil I. Brettis. https://www.brettis.com/Tutorial/08Transformadores.pdf.

[5] Yu W., Xie H. (2012). A review on nanofluids: Preparation, stability mechanisms, and applications. J. Nanomater..

[6] ISO. https://www.iso.org/obp/ui/#iso:std:iso:ts:80004:-2:ed-1:v1:en.

[7] Du B., Li J., Wang B., Xiang J., Zhang Z. Influence of Water Content on the Electrical Properties of Insulating Vegetable Oil-Based Nanofluids. Proceedings of the IEEE Electrical Insulation Conference (EIC).

[8] Nkurikiyimfura I., Wang Y., Pan Z. (2013). Effect of chain-like magnetite nanoparticle aggregates on thermal conductivity of magnetic nanofluid in magnetic field. Exp. Therm. Fluid Sci..

[9] Segal V., Rabinovich A., Nattrass D., Raj K., Nunes A. (2000). Experimental study of magnetic colloidal fluids behavior in power transformers. J. Magn. Magn. Mater..

[10] Sima W., Shi J., Yang Q., Huang S., Cao X. (2015). Effects of conductivity and permittivity of nanoparticle on transformer oil insulation performance: Experiment and theory. IEEE Trans. Dielectr. Electr. Insul..

[11] Karthik R., Cavallini A., Azcarraga C.G. (2014). Investigations on the effect of nanoparticles in mineral oil. Proceedings of the IEEE Conference on Electrical Insulation and Dielectric Phenomena (CEIDP).

[12] Jin H., Andritsch T., Morshuis P.H.F., Smit J.J. AC breakdown voltage and viscosity of mineral oil based fullerene nanofluids. Proceedings of the IEEE Conference on Electrical Insulation and Dielectric Phenomena (CEIDP).

[13] Kopčanský P., Tomčo L., Marton K., Koneracká M., Timko M., Potočová I. (2005). The DC dielectric breakdown strength of magnetic fluids based on transformer oil. J. Magn. Magn. Mater..

[14] Given M.J., Wilson M.P., McGlone P., Timoshkin I.V., Wang T., MacGregor S.J., Lehr J.M. The influence of magnetite nano particles on the behaviour of insulating oils for pulse power applications. Proceedings of the IEEE Conference on Electrical Insulation and Dielectric Phenomena (CEIDP).

[15] Lv Y., Du Y., Zhou J., Li X., Chen M., Li C., Wang G.L. (2012). Nanoparticle Effect on Electrical Properties of Aged Mineral Oil Based Nanofluids.

[16] Timko M., Kopcansky P., Marton K., Tomco L., Holotescu S., Stoian F., Vekas L. Magnetic fluid as cooling and insulation medium for high power transformers. Proceedings of the WSEAS International Conference on Energy, Environment, Ecosystems and Sustainable Development (EEESD).

[17] Lee J.C., Seo H.S., Kim Y.J. (2012). The increased dielectric breakdown voltage of transformer oil-based nanofluids by an external magnetic field. Int. J. Therm. Sci..

[18] Tomčo L., Marton K., Herchl F., Kopčanský P., Potočová I., Koneracká M., Timko M. (2006). The DC and AC insulating properties of magnetic fluids based on transformer oil. Phys. Status Solidi C.

[19] Du B., Li J., Wang B.-M., Zhang Z.-T. Preparation and breakdown strength of Fe_3_O_4_ nanofluid based on transformer oil. Proceedings of the IEEE International Conference on High Voltage Engineering and Application (ICHVE).

[20] Viali W.R., Alcantara G.B., Sartoratto P.P.C., Soler M.A.G., Mosiniewicz-Szablewska E., Andrzejewski B., Morais P.C. (2010). Investigation of the molecular surface coating on the stability of insulating magnetic oils. J. Phys. Chem. C.

[21] Peppas G.D., Bakandritsos A., Charalampakos V.P., Pyrgioti E.C., Tucek J., Zboril R., Gonos I.F. (2016). Ultrastable Natural Ester-Based Nanofluids for High Voltage Insulation Applications. ACS Appl. Mater. Interfaces.

[22] Li W., Zou C., Li X. (2017). Thermo-physical properties of waste cooking oil-based nanofluids. Appl. Therm. Eng..

[23] Jovanović S., Spreitzer M., Tramšek M., Trontelj Z., Suvorov D. (2014). Effect of oleic acid concentration on the physicochemical properties of cobalt ferrite nanoparticles. J. Phys. Chem. C.

[24] Wang L., Dang N., Zhou R., Zhao J., Gao J. Streamer characteristics of TiO_2_ nanofluid/pressboard system with different nanoparticle size. Proceedings of the 16th IET International Conference on AC and DC Power Transmission (ACDC 2020).

[25] Lv Y., Li C., Sun Q., Huang M., Li C., Qi B. (2016). Effect of Dispersion Method on Stability and Dielectric Strength of Transformer Oil-Based TiO2 Nanofluids. Nanoscale Res. Lett..

[26] Fontes D.H., Ribatski G., Bandarra Filho E.P. (2015). Experimental evaluation of thermal conductivity, viscosity and breakdown voltage AC of nanofluids of carbon nanotubes and diamond in transformer oil. Diam. Relat. Mater..

[27] Hwang Y., Park H.S., Lee J.K., Jung W.H. (2006). Thermal conductivity and lubrication characteristics of nanofluids. Curr. Appl. Phys..

[28] Cavallini A., Karthik R., Negri F. (2015). The effect of magnetite, graphene oxide and silicone oxide nanoparticles on dielectric withstand characteristics of mineral oil. IEEE Trans. Dielectr. Electr. Insul..

[29] Li S., Karlsson M., Liu R., Ahniyaz A., Fornara A., Salazar-Sandoval E.J. The Effect of Ceria Nanoparticles on the Breakdown Strength of Transformer Oil. Proceedings of the IEEE International Conference on the Properties and Applications of Dielectric Materials (ICPADM).

[30] Jin H., Andritsch T., Tsekmes I.A., Kochetov R., Morshuis P.H.F., Smit J.J. Thermal Conductivity of Fullerene and TiO_2_ Nanofluids. Proceedings of the IEEE Conference on Electrical Insulation and Dielectric Phenomena (CEIDP).

[31] Jin H., Morshuis P., Mor A.R., Smit J.J., Andritsch T. (2015). Partial Discharge Behavior of Mineral Oil based Nanofluids. IEEE Trans. Dielectr. Electr. Insul..

[32] Jin H., Morshuis P.H.F., Mor A.R., Andritsch T. (2014). An investigation into the dynamics of partial discharge propagation in mineral oil based nanofluids. Proceedings of the IEEE International Conference on Dielectric Liquids (ICDL).

[33] Kúdelčík J., Bury P., Drga J., Kopčanský P., Závišová V., Timko M. The anisotropy of transformer oil-based magnetic fluids studied by acoustic spectroscopy. Proceedings of the ELEKTRO; Faculty of Electrical Engineering.

[34] Pislaru-Danescu L., Morega A.M., Telipan G., Morega M., Dumitru J.B., Marinescu V. (2013). Magnetic nanofluid applications in electrical engineering. IEEE Trans. Magn..

[35] Lv Y., Wang W., Ma K., Zhang S., Zhou Y., Li C., Wang Q. Nanoparticle Effect on Dielectric Breakdown Strength of Transformer Oil-Based Nanofluids. Proceedings of the IEEE Conference on Electrical Insulation and Dielectric Phenomena (CEIDP).

[36] Lv Y., Li X., Du Y., Wang F., Li C. Preparation and breakdown strength of TiO_2_ fluids based on transformer oil. Proceedings of the IEEE Conference on Electrical Insulation and Dielectric Phenomena (CEIDP).

[37] Du Y., Lv Y., Zhou J., Li X., Li C. Breakdown properties of transformer oil-based TiO_2_ nanofluid. Proceedings of the IEEE Conference on Electrical Insulation and Dielectric Phenomena (CEIDP).

[38] Kúdelčík J., Bury P., Kopčanský P., Timko M. Dielectric breakdown in mineral oil ITO 100 based magnetic fluid. Proceedings of the Physics Procedia—International Conference on Magnetic Fluids (ICMF).

[39] Imani M.T., Miethe J.F., Werle P., Bigall N.C., Borsi H. Engineering of multifunctional nanofluids for insulation systems of high voltage apparatus. Proceedings of the IEEE Conference on Electrical Insulation and Dielectric Phenomena (CEIDP).

[40] Liu R., Pettersson L.A.A., Auletta T., Hjortstam O. Fundamental research on the application of nano dielectrics to transformers. Proceedings of the IEEE Conference on Electrical Insulation and Dielectric Phenomena (CEIDP).

[41] Du Y., Lv Y., Wang F., Li X., Li C. Effect of TiO2 nanoparticles on the breakdown strength of transformer oil. Proceedings of the IEEE International Symposium on Electrical Insulation (ISEI).

[42] Lv Y., Wang L., Li X., Du Y., Zhou J., Li C. Experimental investigation of breakdown strength of mineral oil-based nanofluids. Proceedings of the IEEE International Conference on Dielectric Liquids (ICDL).

[43] Lv Y., Zhou Y., Li C., Ma K., Wang Q., Wang W., Zhang S., Jin Z. (2014). Nanoparticle effects on creeping flashover characteristics of oil/pressboard interface. IEEE Trans. Dielectr. Electr. Insul..

[44] Zhou Y., Zhong Y., Chen M., Zhang S., Du Y., Lv Y., Li C., Liu T. Effect of nanoparticles on electrical characteristics of transformer oil-based nanofluids impregnated pressboard. Proceedings of the IEEE International Symposium on Electrical Insulation (ISEI).

[45] Rafiq M., Wang W., Ma K., Zhou Y., Wang Q., Li C., Lv Y. (2014). Insulating and aging properties of transformer oil-based TiO2 nanofluids. Proceedings of the IEEE Conference on Electrical Insulation and Dielectric Phenomena (CEIDP).

[46] Hu Z.F., Ma K., Wang W., Rafiq M., Zhou Y., Wang Q., Du Y., Li C., Lv Y. Thermal aging properties of transformer oil-based TiO2 nanofluids. Proceedings of the IEEE International Conference on Dielectric Liquids (ICDL).

[47] Chen M., Du Y., Lv Y., Zhou J., Li X., Li C. Effect of nanoparticles on the dielectric strength of aged transformer oil. Proceedings of the Conference on Electrical Insulation and Dielectric Phenomena (CEIDP).

[48] Primo V.A., Pérez-Rosa D., García B., Cabanelas J.C. (2019). Evaluation of the Stability of Dielectric Nanofluids for Use in Transformers under Real Operating Conditions. Nanomaterials.

[49] Olmo C., Mendez C., Ortiz F., Delgado F., Valiente R., Werle P. (2019). Maghemite Nanofluid Based on Natural Ester: Cooling and Insulation Properties Assessment. IEEE Access.

[50] Maiti P.K., Chakraborty M. Dissolved Gas Analysis of Thermally Aged Mineral Oil and Vegetable Oil Based Nanofluids. Proceedings of the IEEE International Conference on Properties and Applications of Dielectric Materials 2021.

[51] Oparanti S.O., Khaleed A.A., Abdelmalik A.A. (2021). Nanofluid from Palm Kernel Oil for High Voltage Insulation. Mater. Chem. Phys..

[52] Khaled U., Beroual A. (2020). DC breakdown voltage of natural ester oil-based Fe_3_O_4_, Al_2_O_3_ and SiO_2_ nanofluids. Alex. Eng. J..

[53] Mahidhar G.D.P., Sarathi R., Taylor N., Edin H. (2020). Dielectric properties of silica based synthetic ester nanofluid. IEEE Trans. Dielectr. Electr. Insul..

[54] Ge Y., Xiang L., Li Y., He R., Liu W., Li C. Effect of surface modification of nanomaterial on insulation performance for HVDC transformer. Proceedings of the 7th IEEE International Conference on High Voltage Engineering and Application, ICHVE 2020.

[55] Du B., Liu Q., Shi Y., Zhao Y. (2020). The effect of Fe_3_O_4_ nanoparticle size on electrical properties of nanofluid impregnated paper and trapping analysis. Molecules.

[56] Rajňák M., Kurimský J., Cimbala R., Čonka Z., Bartko P., Šuga M., Paulovičová K., Tóthová J., Karpets M., Kopčanský P. (2020). Statistical analysis of AC dielectric breakdown in transformer oil-based magnetic nanofluids. J. Mol. Liq..

[57] Tripathy S.K., Kumar M.M. (2020). Impact of concentration of nanoparticles on characteristics of transformer oil. J. Nano- Electron. Phys..

[58] Paulovičová K., Tóthová J., Rajňák M., Timko M., Kopčanský P., Lisý V. (2020). Nanofluid Based on New Generation Transformer Oil: Synthesis and Flow Properties. Acta Phys. Pol. A.

[59] Imani M.T., Zámbó D., Miethe J., Werle P., Bigall N.C. (2020). On the dielectrical, electrical and thermo-physical properties of magnetite nanoparticle-doped synthetic ester. Lect. Notes Electr. Eng..

[60] Olmo C., Méndez C., Ortiz F., Delgado F., Ortiz A. (2020). Titania nanofluids based on natural ester: Cooling and insulation properties assessment. Nanomaterials.

[61] Choi C., Yoo H.S., Oh J.M. (2008). Preparation and heat transfer properties of nanoparticle-in-transformer oil dispersions as advanced energy-efficient coolants. Curr. Appl. Phys..

[62] Imani M.T., Werle P., Miethe J.F., Bigall N.C. (2017). Magnetite nanofluid as alternative for conventional insulating liquids. Proceedings of the IEEE International Conference on Dielectric Liquids (ICDL).

[63] Miao J., Dong M., Shen L.-P. A modified electrical conductivity model for insulating oil-based nanofluids. Proceedings of the IEEE International Conference on Condition Monitoring and Diagnosis (CMD).

[64] Jin H., Andritsch T., Tsekmes I.A., Kochetov R., Morshuis P.H.F., Smit J.J. (2014). Properties of Mineral Oil based Silica Nanofluids. IEEE Trans. Dielectr. Electr. Insul..

[65] Liu J., Zhou L., Wu G., Zhao Y., Liu P., Peng Q. (2012). Dielectric frequency response of oil-paper composite insulation modified by nanoparticles. IEEE Trans. Dielectr. Electr. Insul..

[66] Li J., Zhang Z., Zou P., Grzybowski S., Zahn M. (2012). Preparation of a vegetable oil-based nanofluid and investigation of its breakdown and dielectric properties. IEEE Electr. Insul. Mag..

[67] Ghasemi J., Jafarmadar S., Nazari M. (2015). Effect of magnetic nanoparticles on the lightning impulse breakdown voltage of transformer oil. J. Magn. Magn. Mater..

[68] Emara M.M., Mansour D.E.A., Azmy A.M. (2015). Dielectric properties of aged mineral oil filled with TiO_2_ nanoparticles. Proceedings of the International Conference on Electric Power and Energy Conversion Systems (EPECS).

[69] Pislaru-Danescu L., Morega A.M., Morega M., Stoica V., Marinica O.M., Nouras F., Paduraru N., Borbath I., Borbath T. (2013). Prototyping a Ferrofluid-Cooled Transformer. IEEE Trans. Ind. Appl..

[70] Becerra M., Aljure M., Pourrahimi A.M., Roman F. (2021). High field conduction in mineral oil based zno nanofluids prior to negative streamer inception. J. Phys. Commun..

[71] Chen Y., Luo P., He D., Ma R. (2021). Numerical simulation and analysis of natural convective flow and heat transfer of nanofluid under electric field. Int. Commun. Heat Mass Transf..

[72] Atiya E.G., Mansour D.E.A., Izzularab M.A. (2020). Partial discharge development in oil-based nanofluids: Inception, propagation and time transition. IEEE Access.

[73] Liu D., Zhou Y., Yang Y., Zhang L., Jin F. (2016). Characterization of high performance AIN nanoparticle-based transformer oil nanofluids. IEEE Trans. Dielectr. Electr. Insul..

[74] Hanai M., Hosomi S., Kojima H., Hayakawa N., Okubo H. Dependence of TiO2 and ZnO nanoparticle concentration on electrical insulation characteristics of insulating oil. Proceedings of the IEEE Conference on Electrical Insulation and Dielectric Phenomena (CEIDP).

[75] Zhang Z., Li J., Zou P., Grzybowski S. Electrical properties of nano-modified insulating vegetable oil. Proceedings of the IEEE Conference on Electrical Insulation and Dielectric Phenomena (CEIDP).

[76] Taghikhani Z., Taghikhani M.A., Gharehpetian G.B. (2021). Mineral oil based CuO nanofluid-immersed transformers analysis concerning the efficacy of nanocrystalline alloy core in reduction of losses and HST. J. Magn. Magn. Mater..

[77] Taghikhani Z., Taghikhani M.A., Gharehpetian G.B. (2021). A comprehensive investigation on the efficiency of alumina nanoparticles in ONAN and OFAN cooling performance enhancement of transformers. Powder Technol..

[78] Muangpratoom P. (2021). The effect of temperature on the electrical characteristics of nanofluids based on palm oil. J. Eng. Technol. Sci..

[79] Koutras K.N., Naxakis I.A., Pyrgioti E.C., Charalampakos V.P., Gonos I.F., Antonelou A.E., Yannopoulos S.N. (2020). The Influence of Nanoparticles ’ Conductivity and Charging on Dielectric Properties of Ester Oil Based Nanofluid. Energies.

[80] Du B.X., Li X.L. (2014). High thermal conductivity transformer oil filled with BN nanoparticles. Proceedings of the IEEE International Conference on Dielectric Liquids (ICDL).

[81] Prasath R.T.A.R., Karthik R., Iruthayarajan M.W. Enhancement of critical properties of pure and aged transformer oil using nanocomposites. Proceedings of the International Conference on Circuits, Power and Computing Technologies (ICCPCT).

[82] Dong M., Shen L.P., Wang H., Wang H.B., Miao J. (2013). Investigation on the Electrical Conductivity of Transformer Oil-Based AlN Nanofluid. J. Nanomater..

[83] Wang Q., Rafiq M., Lv Y., Li C., Yi K. (2016). Preparation of three types of transformer oil-based nanofluids and comparative study on the effect of nanoparticle concentrations on insulating property of transformer oil. J. Nanotechnol..

[84] Peppas G.D., Charalampakos V.P., Pyrgioti E.C., Danikas M.G., Bakandritsos A., Gonos I.F. (2016). Statistical investigation of AC breakdown voltage of nanofluids compared with mineral and natural ester oil. IET Sci. Meas. Technol..

[85] Mohamad N.A., Azis N., Jasni J., Mohd Zainal M.Z.A., Yunus R., Yaakub Z. (2020). Effect of surfactants on the lightning breakdown voltage of palm oil and coconut oil based Al_2_O_3_ nanofluids. Nanotechnology.

[86] Fernández I., Valiente R., Ortiz F., Renedo C.J., Ortiz A. (2020). Effect of TiO_2_ and ZnO Nanoparticles on the Performance of Dielectric Nanofluids Based on Vegetable Esters During Their Aging. Nanomaterials.

[87] Xuan Y., Li Q. (2000). Heat transfer enhancement of nanofluids. Int. J. Heat Fluid Flow.

[88] Chiesa M., Das S.K. (2009). Experimental investigation of the dielectric and cooling performance of colloidal suspensions in insulating media. Colloids Surf. A Physicochem. Eng. Asp..

[89] Atiya E.G., Mansour D.E.A., Khattab R.M., Azmy A.M. (2015). Dispersion behavior and breakdown strength of transformer oil filled with TiO_2_ nanoparticles. IEEE Trans. Dielectr. Electr. Insul..

[90] Mansour D.E.A., Atiya E.G., Khattab R.M., Azmy A.M. Effect of titania nanoparticles on the dielectric properties of transformer oil-based nanofluids. Proceedings of the IEEE Conference on Electrical Insulation and Dielectric Phenomena (CEIDP).

[91] Khan S.A., Khan A.A., Tariq M. Performance Evaluation of Nano-fluids Based Mineral Oils for Application in Power Transformers. Proceedings of the 2020 3rd International Conference on Energy, Power and Environment (ICEPE).

[92] Maneerat N., Makmork K., Kittikhuntharadol Y., Suksai N., Chusang T., Pattanadech N. AC Breakdown and Resistivity of Natural Ester Based Nanofluids. Proceedings of the 8th International Conference on Condition Monitoring and Diagnosis, CMD 2020.

[93] Koutras K., Pyrgioti E., Naxakis I., Peppas G., Charalampakos V., Gonos I. A Comparative Study on Dielectric Properties of Untreated and Surface-modified Natural Ester Based Nanofluids. Proceedings of the 7th IEEE International Conference on High Voltage Engineering and Application, ICHVE 2020.

[94] Karthik R., Raja T., Madavan R. (2013). Enhancement of Critical Characteristics of Transformer Oil Using Nanomaterials. Arab. J. Sci. Eng..

[95] Hussin N., Subri N.A., Azizie N.A., Khalil A.N.M., Jamil M.K.M., Abd-Rahman R., Arshad S.N.M. (2021). Low Concentration Vegetable Oil Based Nanofluid: Dielectric properties, AC Breakdown Voltage and Kinematic Viscosity. J. Phys. Conf. Ser..

[96] Rafiq M., Lv Y., Li C., Sun Q. (2020). Effect of Al_2_O_3_ nanorods on the performance of oil-impregnated pressboard insulation. Electr. Eng..

[97] Sens M.A., Ueti E., Filho F.A., Matt C.F.T., Polasek A., Furtado J.G.M., Da Silva L.A.F., Guedes V.G., Lima W.F., Garcia R.W.S. Electromagnetic characterization of Magnetic Nanofluid. Proceedings of the Conference on Precision Electromagnetic Measurements (CPEM).

[98] Jin H., Andritsch T., Morshuis P.H.F., Smit J.J. AC Breakdown Voltage and Viscosity of Mineral Oil based SiO_2_ Nanofluids. Proceedings of the IEEE Conference on Electrical Insulation and Dielectric Phenomena (CEIDP).

[99] Zhou J., Du Y., Chen M., Li C., Li X., Lv Y. AC and lightning breakdown strength of transformer oil modified by semiconducting nanoparticles. Proceedings of the IEEE Conference on Electrical Insulation and Dielectric Phenomena (CEIDP).

[100] Ma K., Lv Y., Wang W., Zhou Y., Zhang S., Li C. Influence of semiconductive nanoparticle on sulfur corrosion behaviors in oil-paper insulation. Proceedings of the IEEE Conference on Electrical Insulation and Dielectric Phenomena (CEIDP).

[101] Lv Y., Zhang S., Du Y., Chen M., Li C. (2013). Effect of oleic acid surface modification on dispersibility of TiO_2_ nanoparticles in transformer oils. Wuji Cailiao Xuebao/J. Inorg. Mater..

[102] Cimbala R., Király J., German-Sobek M., Pavlik M. (2014). Dielectric spectroscopy of transformer oil based ferrofluid from view of i(t) characteristics at initial stage of ageing. Proceedings of the International Scientific Conference on Electric Power Engineering (EPE).

[103] Du Y., Lv Y., Zhou J., Chen M., Li X., Li C. Effect of ageing on insulating property of mineral Oil-based TiO_2_ nanofluids. Proceedings of the IEEE International Conference on Dielectric Liquids (ICDL).

[104] Du Y., Lv Y., Li C., Chen M., Zhong Y., Zhou J., Li X., Zhou Y. (2012). Effect of semiconductive nanoparticles on insulating performances of transformer oil. IEEE Trans. Dielectr. Electr. Insul..

[105] Du Y., Lv Y., Li C., Chen M., Zhou J., Li X., Zhou Y., Tu Y. (2011). Effect of electron shallow trap on breakdown performance of transformer oil-based nanofluids. J. Appl. Phys..

[106] Du Y., Lv Y., Li C., Zhong Y., Chen M., Zhang S., Zhou Y., Chen Z. (2012). Effect of water adsorption at nanoparticle-oil interface on charge transport in high humidity transformer oil-based nanofluid. Colloids Surf. A Physicochem. Eng. Asp..

[107] Maity D., Agrawal D.C. (2007). Synthesis of iron oxide nanoparticles under oxidizing environment and their stabilization in aqueous and non-aqueous media. J. Magn. Magn. Mater..

[108] Du B., Li J., Wang F., Yao W., Yao S. (2015). Influence of Monodisperse Fe_3_O_4_ Nanoparticle Size on Electrical Properties of Vegetable Oil-Based Nanofluids. J. Nanomater..

[109] Li J., Du B., Wang F., Yao W., Yao S. (2016). The effect of nanoparticle surfactant polarization on trapping depth of vegetable insulating oil-based nanofluids. Phys. Lett. Sect. A Gen. At. Solid State Phys..

[110] Mansour D.E.A., Elsaeed A.M. Heat transfer properties of transformer oil-based nanofluids filled with Al_2_O_3_ nanoparticles. Proceedings of the IEEE International Conference on Power and Energy (PECon).

[111] Pugazhendhi Sugumaran C. Experimental evaluation on dielectric and thermal characteristics of nano filler added transformer oil. Proceedings of the IEEE International Conference on High Voltage Engineering and Application (ICHVE).

[112] Mergos J.A., Athanassopoulou M.D., Argyropoulos T.G., Dervos C.T. (2012). Dielectric properties of nanopowder dispersions in paraffin oil. IEEE Trans. Dielectr. Electr. Insul..

[113] Ali R.H.M., Alam M.F., Khan K.A., Muzaffar S. Thermo-electric Impact of Nano-Materials on Transformer Oil and Synthetic Ester Oil. Proceedings of the 2020 IEEE 10th International Conference on “Nanomaterials: Applications and Properties”, NAP 2020.

[114] Zhong Y., Lv Y., Li C., Du Y., Chen M., Zhang S., Zhou Y., Chen L. (2013). Insulating Properties and Charge Characteristics of Natural Ester Fluid Modified by TiO2 Semiconductive Nanoparticles. IEEE Trans. Dielectr. Electr. Insul..

[115] Huang Z., He G., Li J., Wang F., Zhang R., Yao D. (2021). Exponentially reduced carrier mobility of natural ester via blocking effect of 2D hexagonal boron nitride nanosheets. High Volt..

[116] Dhar P., Katiyar A., Maganti L.S., Pattamatta A., Das S.K. (2016). Superior dielectric breakdown strength of graphene and carbon nanotube infused nano-oils. IEEE Trans. Dielectr. Electr. Insul..

[117] Krishna Kumar P., Senthil Kumar S., Ravindran M. Investigation on mixed insulating fluids with nano fluids and antioxidants. Proceedings of the International Conference on Advances in Electrical Engineering (ICAEE).

[118] Ravi Babu S., Saras Chandra A., Ramesh Babu P. (2020). Experimental investigation of free connective heat transfer augmentation using transformer oil-Al_2_O_3_ nanofluid. IOP Conference Series: Materials Science and Engineering.

[119] Moghanlou F.S., Khorrami S.A., Esmaeilzadeh E., Vajdi M. (2021). Effect of strong electric field on heat transfer enhancement in a mini channel containing—Al_2_O_3_/oil nanofluid. J. Braz. Soc. Mech. Sci. Eng..

[120] Nazari M., Rasoulifard M.H., Hosseini H. (2016). Dielectric breakdown strength of magnetic nanofluid based on insulation oil after impulse test. J. Magn. Magn. Mater..

[121] Bakrutheen M., Karthik R., Madavan R. Investigation of Critical Parameters of Insulating Mineral Oil Using Semiconductive Nanoparticles. Proceedings of the International Conference on Circuits, Power and Computing Technologies (ICCPCT).

[122] Lee J.-C., Lee W.-H., Lee S.-H., Lee S. (2012). Positive and negative effects of dielectric breakdown in transformer oil based magnetic fluids. Mater. Res. Bull..

[123] Stoian F.D., Holotescu S., Taculescu A., Marinica O., Resiga D., Timko M., Kopčanský P., Rajnak M. Characteristic properties of a magnetic nanofluid used as cooling and insulating medium in a power transformer. Proceedings of the International Symposium on Advanced Topics in Electrical Engineering (ATEE).

[124] Chitra S.R., Sendhilnathan S. (2016). Experimental Investigations on Dielectric Fluids Behavior in High-Power Transformers. Int. J. Appl. Ceram. Technol..

[125] Tangthieng C., Finlayson B.A., Maulbetsch J., Cader T. (1999). Heat transfer enhancement in ferrofluids subjected to steady magnetic fields. J. Magn. Magn. Mater..

[126] Ramu T.S., Keshavan B.K., Balasubramanya Murthy K.N. Application of a class of nano fluids to improve the loadability of power transformers. Proceedings of the IEEE International Conference on Properties and Applications of Dielectric Materials (ICPADM).

[127] Almeida C., Paul S., Asirvatham L.G., Manova S., Nimmagadda R., Bose J.R., Wongwises S. (2020). Experimental studies on thermophysical and electrical properties of graphene-transformer oil nanofluid. Fluids.

[128] Sarov Mohan S., Preetha P. Optimization of Filler Loading of Multi-Particle Mineral Oil Nanofluid for Transformer Insulation. Proceedings of the 2020 IEEE 3rd International Conference on Dielectrics (ICD).

[129] Farade R.A., Wahab N.I.A., Mansour D.E.A., Azis N.B., Jasni J.B., Soudagar M.E.M., Siddappa V. (2020). Development of graphene oxide-based nonedible cottonseed nanofluids for power transformers. Materials.

[130] Taha-Tijerina J., Narayanan T.N., Gao G., Rohde M., Tsentalovich D.A., Pasquali M., Ajayan P.M. (2012). Electrically insulating thermal nano-oils using 2D fillers. ACS Nano.

[131] Du B.X., Li X.L., Li J. (2015). Thermal conductivity and dielectric characteristics of transformer oil filled with bn and Fe_3_O_4_ nanoparticles. IEEE Trans. Dielectr. Electr. Insul..

[132] Li J., Liao R., Yang L. Investigation of natural ester based liquid dielectrics and nanofluids. Proceedings of the IEEE International Conference on High Voltage Engineering and Application (ICHVE).

[133] Lv Y., Rafiq M., Li C., Shan B. (2017). Study of dielectric breakdown performance of transformer oil based magnetic nanofluids. Energies.

[134] Irwanto, Azcarraga C.G., Suwarno, Cavallini A., Negri F. Ferrofluid effect in mineral oil: PDIV, streamer, and breakdown voltage. Proceedings of the IEEE International Conference on High Voltage Engineering and Application (ICHVE).

[135] Rafiq M., Li C., Khan I., Zhifeng H., Lv Y., Yi K. Preparation and Breakdown Properties of Mineral Oil Based Alumina Nanofluids. Proceedings of the International Conference on Emerging Technologies (ICET).

[136] Rafiq M., Li C., Ge Y., Lv Y., Yi K. (2016). Effect of Fe_3_O_4_ nanoparticle concentrations on dielectric property of transformer oil. Proceedings of the IEEE International Conference on High Voltage Engineering and Application (ICHVE).

[137] Pislaru-Danescu L., Morega A., Telipan G., Stoica V. (2010). Nanoparticles of ferrofluid Fe_3_O_4_ synthetised by coprecipitation method used in microactuation process. Optoelectron. Adv. Mater. Rapid Commun..

[138] Morega A.M., Morega M., Pislaru-Danescu L., Stoica V., Nouras F., Stoian F.D. A novel, ferrofluid-cooled transformer. electromagnetic field and heat transfer by numerical simulation. Proceedings of the International Conference on Optimization of Electrical and Electronic Equipment (OPTIM).

[139] Zhang L., He R., Gu H.C. (2006). Oleic acid coating on the monodisperse magnetite nanoparticles. Appl. Surf. Sci..

[140] Rafiq M., Khan D., Ali M. Dielectric Properties of Transformer Oil based Silica Nanofluids. Proceedings of the Power Generation System and Renewable Energy Technologies (PGSRET).

[141] Lv Y., Zhou Y., Li C., Wang Q., Qi B. (2014). Recent progress in nanofluids based on transformer oil: Preparation and electrical insulation properties. IEEE Electr. Insul. Mag..

[142] Nasiri A., Shariaty-Niasar M., Rashidi A., Amrollahi A., Khodafarin R. (2011). Effect of dispersion method on thermal conductivity and stability of nanofluid. Exp. Therm. Fluid Sci..

[143] Aberoumand S., Jafarimoghaddam A., Moravej M., Aberoumand H., Javaherdeh K. (2016). Experimental study on the rheological behavior of silver-heat transfer oil nanofluid and suggesting two empirical based correlations for thermal conductivity and viscosity of oil based nanofluids. Appl. Therm. Eng..

[144] Daraio H., Jin S. (2012). Synthesis and Patterning Methods for Nanostructures Useful for Biological Applications. Nanotechnology for Biology and Medicine.

[145] Thapa D., Palkar V.R., Kurup M.B., Malik S.K. (2004). Properties of magnetite nanoparticles synthesized through a novel chemical route. Mater. Lett..

[146] Jia B., Gao L. (2007). Fabrication of “tadpole”-like magnetite/multiwalled carbon nanotube heterojunctions and their self-assembly under external magnetic field. J. Phys. Chem. B.

[147] Tsai C.-W., Langner E.H.G. (2016). The effect of synthesis temperature on the particle size of nano-ZIF-8. Microporous Mesoporous Mater..

[148] Hong R., Pan T., Qian J., Li H. (2006). Synthesis and surface modification of ZnO nanoparticles. Chem. Eng. J..

[149] Do Kim K., Kim S.S., Choa Y.-H., Kim H.T. (2007). Formation and Surface Modification of Fe_3_O_4_ Nanoparticles by Co-precipitation and Sol-gel Method. J. Ind. Eng. Chem.

[150] Tsai T.H., Kuo L.S., Chen P.H., Lee D.S., Yang C.T. (2010). Applications of ferro-nanofluid on a micro-transformer. Sensors.

[151] Khalil M., Yu J., Liu N., Lee R.L. (2014). Non-aqueous modification of synthesized hematite nanoparticles with oleic acid. Colloids Surf. A Physicochem. Eng. Asp..

[152] Yamaura M., Camilo R.L., Sampaio L.C., Macêdo M.A., Nakamura M., Toma H.E. (2004). Preparation and characterization of (3-aminopropyl)triethoxysilane-coated magnetite nanoparticles. J. Magn. Magn. Mater..

[153] Amici J., Allia P., Tiberto P., Sangermano M. (2011). Poly(ethylene glycol)-coated Fe_3_O_4_ nanoparticles by UV-thiol-ene addition of PEG dithiol on vinyl-functionalized magnetite surface. Macromol. Chem. Phys..

[154] Koneracká M., Kopčanský P., Antalík M., Timko M., Ramchand C.N., Lobo D., Mehta R.V., Upadhyay R.V. (1999). Immobilization of proteins and enzymes to fine magnetic particles. J. Magn. Magn. Mater..

[155] Liu X., Ma Z., Xing J., Liu H. (2004). Preparation and characterization of amino-silane modified superparamagnetic silica nanospheres. J. Magn. Magn. Mater..

[156] Nkurikiyimfura I., Wang Y., Pan Z., Hu D. Enhancement of thermal conductivity of magnetic nanofluids in magnetic field. Proceedings of the International Conference on Materials for Renewable Energy and Environment (ICMREE).

[157] Bourlinos A.B., Bakandritsos A., Georgakilas V., Petridis D. (2002). Surface modification of ultrafine magnetic iron oxide particles. Chem. Mater..

[158] Van Ewijk G.A., Vroege G.J., Philipse A.P. (1999). Convenient preparation methods for magnetic colloids. J. Magn. Magn. Mater..

[159] Zhou W., Zhou Z., Zhang L., Guo L., Du Y., Lv Y. (2010). Seed-mediated synthesis and characterization of Ni flower-like nanomaterials. J. Nanosci. Nanotechnol..

[160] Shojaie A.F., Loghmani M.H. (2010). La3+ and Zr4+ co-doped anatase nano TiO_2_ by sol-microwave method. Chem. Eng. J..

[161] Zhou J., Song B., Zhao G., Han G. (2012). Effects of acid on the microstructures and properties of three-dimensional TiO_2_ hierarchical structures by solvothermal method. Nanoscale Res. Lett..

[162] Talaei Z., Rashidi A.M., Amrollahi A., Mahjoub A.R. The effect of carboxylic group concentration on the stability and thermal conductivity of carbon nanotub fluid as heat transfer media. Proceedings of the International Vacuum Electron Sources Conference (IVESC) and NANOcarbon.

[163] Teng T.P., Wang W.P., Hsu Y.C. (2016). Fabrication and Characterization of Nanocarbon-Based Nanofluids by Using an Oxygen–Acetylene Flame Synthesis System. Nanoscale Res. Lett..

[164] Li D. Preparation and characterization of lipophilic copper nanoparticle. Proceedings of the International Conference on Remote Sensing, Environment and Transportation Engineering (RSETE).

[165] Yang B., Han Z.H. (2006). Temperature-dependent thermal conductivity of nanorod-based nanofluids. Appl. Phys. Lett..

[166] Vékás L., Bica D., Marinica O. (2006). Magnetic nanofluids stabilized with various chain length surfactants. Rom. Rep. Phys..

[167] Józefczak A. (2009). Study of low concentrated ionic ferrrofluid stability in magnetic field by ultrasound spectroscopy. J. Magn. Magn. Mater..

[168] Sarojini K.G.K., Manoj S.V., Singh P.K., Pradeep T., Das S.K. (2013). Electrical conductivity of ceramic and metallic nanofluids. Colloids Surf. A Physicochem. Eng. Asp..

[169] Masteri-Farahani M., Bahmanyar M., Mohammadikish M. (2012). Organic-inorganic hybrid nanomaterials prepared from 4-formyl benzo-12-crown-4-ether and silica coated magnetite nanoparticles. J. Nanostruct..

[170] Tombácz E., Bica D., Hajdú A., Illés E., Majzik A., Vékás L. (2008). Surfactant double layer stabilized magnetic nanofluids for biomedical application. J. Phys. Condens. Matter.

[171] Kim D., Kwon Y., Cho Y., Li C., Cheong S., Hwang Y., Lee J., Hong D., Moon S. (2009). Convective heat transfer characteristics of nanofluids under laminar and turbulent flow conditions. Curr. Appl. Phys..

[172] Meenakshi K.S., Pradeep Jaya Sudhan E. Preparation and characterization of titanium oxide-water based nanofluids by one step method for heat transfer applications. Proceedings of the International Conference on Nanoscience, Engineering and Technology (ICONSET).

[173] Nguyen C.T., Roy G., Gauthier C., Galanis N. (2007). Heat transfer enhancement using Al_2_O_3_-water nanofluid for an electronic liquid cooling system. Appl. Therm. Eng..

[174] Teng T.-P., Ting C.-H., Chun C.-C. (2019). Characteristics of carbon-based nanofluids and their application in a brazed plate heat exchanger under laminar flow. Appl. Therm. Eng..

[175] Das S.K., Putra N., Thiesen P., Roetzel W. (2003). Temperature dependence of thermal conductivity enhancement for nanofluids. J. Heat Transf..

[176] Chon C.H., Kihm K.D., Lee S.P., Choi S.U.S. (2005). Empirical correlation finding the role of temperature and particle size for nanofluid (Al_2_O_3_) thermal conductivity enhancement. Appl. Phys. Lett..

[177] Murshed S.M.S., Leong K.C., Yang C. Thermal conductivity of nanoparticle suspensions (nanofluids). Proceedings of the IEEE Conference on Emerging Technologies—Nanoelectronics (INEC).

[178] Xie H., Wang J., Xi T., Liu Y., Ai F. (2002). Dependence of the thermal conductivity on nanoparticle-fluid mixture on the base fluid. J. Mater. Sci. Lett..

[179] Wen D., Ding Y. (2006). Natural convective heat transfer of suspensions of titanium dioxide nanoparticles (nanofluids). IEEE Trans. Nanotechnol..

[180] Parekh K., Upadhyay R.V. (2004). Characterization of transformer oil based magnetic fluid. Indian J. Eng. Mater. Sci..

[181] Ettefaghi E., Ahmadi H., Rashidi A., Nouralishahi A., Mohtasebi S.S. (2013). Preparation and thermal properties of oil-based nanofluid from multi-walled carbon nanotubes and engine oil as nano-lubricant. Int. Commun. Heat Mass Transf..

[182] Patel R., Parekh K., Upadhyay R.V., Mehta R.V. (2004). Rheology of transformer oil based ferrofluids. Indian J. Eng. Mater. Sci..

[183] Witharana S., Weliwita J.A. Suspended nanoparticles as a way to improve thermal energy transfer efficiency. Proceedings of the IEEE International Conference on Information and Automation for Sustainability (ICIAFS).

[184] Qiu G., Wang Q., Wang C., Lau W., Guo Y. (2007). Polystyrene/Fe_3_O_4_ magnetic emulsion and nanocomposite prepared by ultrasonically initiated miniemulsion polymerization. Ultrason. Sonochem..

[185] Lee J.H., Hwang K.S., Jang S.P., Lee B.H., Kim J.H., Choi S.U.S., Choi C.J. (2008). Effective viscosities and thermal conductivities of aqueous nanofluids containing low volume concentrations of Al_2_O_3_ nanoparticles. Int. J. Heat Mass Transf..

[186] Lienhard J.H. (1981). A Heat Transfer Textbook.

[187] Keblinski P., Phillpot S.R., Choi S.U.S., Eastman J.A. (2001). Mechanisms of heat flow in suspensions of nano-sized particles (nanofluids). Int. J. Heat Mass Transf..

[188] Hwang J.G., Zahn M., O’Sullivan F.M., Pettersson L.A.A., Hjortstam O., Liu R. (2010). Effects of nanoparticle charging on streamer development in transformer oil-based nanofluids. J. Appl. Phys..

[189] Hwang J.G., O’Sullivan F., Zahn M., Hjortstam O., Pettersson L.A.A., Liu R. Modeling of streamer propagation in transformer oil-based nanofluids. Proceedings of the IEEE Conference on Electrical Insulation and Dielectric Phenomena (CEIDP).

[190] Jin H., Morshuis P.H.F., Smit J.J., Andritsch T. (2014). The effect of surface treatment of silica nanoparticles on the breakdown strength of mineral oil. Proceedings of the IEEE International Conference on Dielectric Liquids (ICDL).

[191] Mendelev V.S., Ivanov A.O. (2004). Ferrofluid aggregation in chains under the influence of a magnetic field. Phys. Rev. E Stat. Nonlinear Soft Matter Phys..

[192] Segal V., Raj K. (1998). An investigation of power transformer cooling with magnetic fluids. Indian J. Eng. Mater. Sci..

[193] Liao R., Liu J., Yang L., Wang K., Hao J., Ma Z., Gao J., Lv Y. (2015). Quantitative analysis of insulation condition of oil-paper insulation based on frequency domain spectroscopy. IEEE Trans. Dielectr. Electr. Insul..

[194] Coulibaly M.-L., Perrier C., Marugan M., Beroual A. (2013). Aging behavior of cellulosic materials in presence of mineral oil and ester liquids under various conditions. IEEE Trans. Dielectr. Electr. Insul..

[195] Lelekakis N., Guo W., Martin D., Wijaya J., Susa D. (2012). A field study of aging in paper-oil insulation systems. IEEE Electr. Insul. Mag..

[196] Chaudhari S., Patil S., Zambare R., Chakraborty S. IEEE Staff; IEEE Staff Exploration on use of ferrofluid in power transformers. Proceedings of the IEEE International Conference on Properties and Applications of Dielectric Materials (ICPADM).

